# Resources to Facilitate Use of the Altered Schaedler Flora (ASF) Mouse Model to Study Microbiome Function

**DOI:** 10.1128/msystems.00293-22

**Published:** 2022-08-15

**Authors:** Alexandra Proctor, Shadi Parvinroo, Tanner Richie, Xinglin Jia, Sonny T. M. Lee, Peter D. Karp, Suzanne Paley, Aleksandar D. Kostic, Joseph F. Pierre, Michael J. Wannemuehler, Gregory J. Phillips

**Affiliations:** a Department of Veterinary Microbiology, Iowa State University, Ames, Iowa, USA; b Division of Biology, Kansas State University, Manhattan Kansas, USA; c Bioinformatics Research Group, SRI International, Menlo Park, California, USA; d Department of Microbiology and Immunology, Joslin Diabetes Center, Harvard University, Cambridge Massachusetts, USA; e Department of Nutritional Sciences, University of Wisconsin-Madison, Madison Wisconsin, USA; Duke University School of Medicine

**Keywords:** ASF, Schaedler, gnotobiotic mice, microbiome function, qPCR, microbiota evolution

## Abstract

Animals colonized with a defined microbiota represent useful experimental systems to investigate microbiome function. The altered Schaedler flora (ASF) represents a consortium of eight murine bacterial species that have been used for more than 4 decades where the study of mice with a reduced microbiota is desired. In contrast to germ-free mice, or mice colonized with only one or two species, ASF mice show the normal gut structure and immune system development. To further expand the utility of the ASF, we have developed technical and bioinformatic resources to enable a systems-based analysis of microbiome function using this model. Here, we highlighted four distinct applications of these resources that enable and improve (i) measurements of the abundance of each ASF member by quantitative PCR; (ii) exploration and comparative analysis of ASF genomes and the metabolic pathways they encode that comprise the entire gut microbiome; (iii) global transcriptional profiling to identify genes whose expression responds to environmental changes within the gut; and (iv) discovery of genetic changes resulting from the evolutionary adaptation of the microbiota. These resources were designed to be accessible to a broad community of researchers that, in combination with conventionally-reared mice (i.e., with complex microbiome), should contribute to our understanding of microbiome structure and function.

**IMPORTANCE** Improved experimental systems are needed to advance our understanding of how the gut microbiome influences processes of the mammalian host as well as microbial community structure and function. An approach that is receiving considerable attention is the use of animal models that harbor a stable microbiota of known composition, i.e., defined microbiota, which enables control over an otherwise highly complex and variable feature of mammalian biology. The altered Schaedler flora (ASF) consortium is a well-established defined microbiota model, where mice are stably colonized with 8 distinct murine bacterial species. To take better advantage of the ASF, we established new experimental and bioinformatics resources for researchers to make better use of this model as an experimental system to study microbiome function.

## INTRODUCTION

It is well established that microorganisms colonizing the gastrointestinal (GI) tract contribute to multiple processes essential for the overall health and well-being of the host, including nutrient acquisition, immune system development, disease processes, as well as neurological function through the gut-brain-axis (1–4). Despite the importance of the microbiome, including as a potential target to prevent and treat disease, a significant barrier to overcome remains the immense complexity of the GI community makes it difficult to reproducibly study its structure and function across time and experimental settings. The mammalian GI microbiome is comprised of hundreds of microbial species and species variants (5–7) that encode large numbers of nonredundant genes (8, 9). Experimental manipulation of the microbiome can also be challenging because many microorganisms cannot be easily cultured *in vitro* (10, 11).

To overcome these challenges, animals colonized with simplified or defined microbial communities are used. For example, the fruit fly (Drosophila melanogaster) and zebrafish (Danio rerio), which are colonized naturally with relatively few microbial species (10), have been developed into microbiome research models. To address questions specific to the mammalian microbiome, mice offer advantages because the anatomy and microbial composition at higher taxonomic classifications are shared with other mammals, including humans (12, 13). Experimental systems include the use of germ-free (GF) mice, which can be colonized with the microbiota from other sources (14) to assess how the microbiome impacts the host (15, 16). GF mice can also be colonized with specific bacterial strains (e.g., mono-associated) to reduce complexity (17, 18). However, these mice are often deficient in many fundamental host processes, such as immune system development and function, GI morphology and function, and colonization resistance (14, 15, 19–21).

To simulate the structure and function of a conventional (CONV) animal’s microbiome more closely, i.e., complex microbiota mice, animals are colonized with defined microbial consortia (14, 19). Importantly, these gnotobiotic mouse models can display growth and developmental phenotypes that more closely resemble that of CONV animals, such as colonization resistance and immune system development (22–24). One of the first gnotobiotic mouse models was established by Russell Schaedler and colleagues, who colonized GF mice with combinations of cultivable aerobic, aerotolerant and strict anaerobic bacterial strains isolated from the mouse GI tract (22–24). A consortium of eight bacterial species known as the “altered Schaedler flora” (ASF) was established by Orcutt et al. (25), which remains widely used today. The ASF represents microorganisms commonly observed in CONV mice (26, 27). Each of the eight species can be cultured *in vitro* and is stably transmitted from the dam to pup over multiple generations (24, 28). The ASF members colonize the entire GI tract in relative proportions, similar to CONV mice (27). Importantly, the ASF also supports the development and function of the mouse immune system and GI tract physiology (24).

While initially used by rodent vendors under contract by the National Cancer Institute (NCI), ASF mice have been used to study the role of the microbiota in microbial pathogenesis (29–34), host immune system development and responses (35–38) and contribution to disease processes (39–41), among others.

Despite its use to address a wide variety of questions, the model has not been extensively leveraged as a system-based approach to interrogating the microbiome function of individual community members (42, 43). To improve the utilization of this model microbial consortium for systems- and functional-level study of the gut microbiome, we reported multiple technical and bioinformatic resources that can be used to improve our mechanistic understanding of microbiome function. To illustrate the application of these resources, we highlighted their use in four specific experimental applications.

## RESULTS AND DISCUSSION

### ASF genome sequences.

To improve on the coverage of ASF genome sequences (44), we generated complete or nearly complete genome sequences for each member. Features of the ASF genomes, along with updated taxonomic classifications are shown in Table 1. The ASF chromosomes have a relatively wide size range; three chromosomes (ASF492, 502, 519) were greater than 6-Mb and three chromosomes (ASF360, 361, 457) were less than 2.5-Mb. Closed chromosomes are now available for five of the ASF members (ASF356, 361, 457, 500, and 502) and the two largest chromosomes are contained within 6 contigs (ASF492, 519), indicating the newly sequenced genomes represent the near-complete coding capacity of the ASF. No evidence of plasmids in the ASF genomes was found. The genome sequences were also used to corroborate the taxonomic assignments of each ASF member (Table 1) using the Type (Strain) Genome Server (TYGS) and TYGS database (45, 46).

**TABLE 1:**
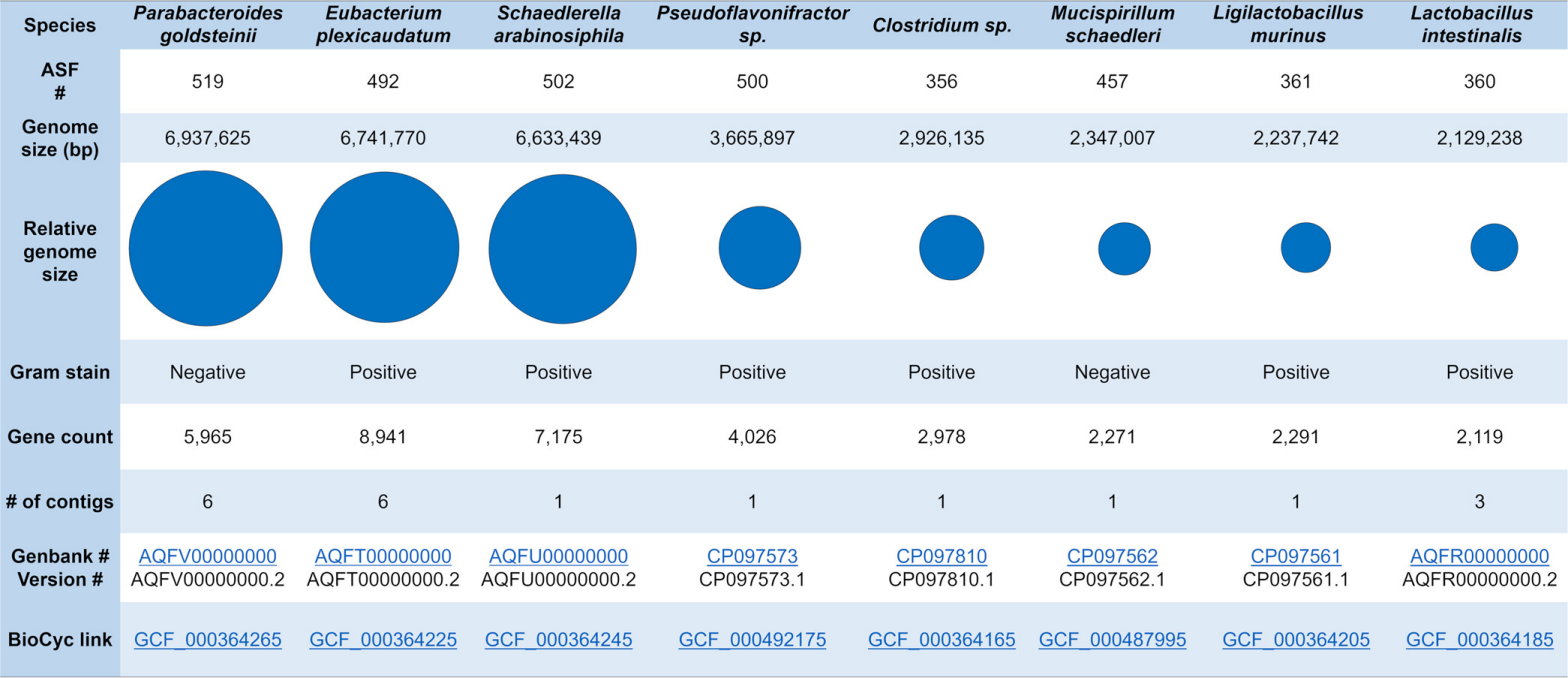
Characteristics of the altered Schaedler flora and their genomes^*a*^

a Genbank Version: AQFV00000000, AQFT00000000, AQFU00000000, CP097573, CP097810, CP097562, CP097561, and AQFR00000000; BioCyc link : GCF_000364265, GCF_000364225, GCF_000364245, GCF_000492175, GCF_000364165, GCF_000487995, GCF_000364205, and GCF_000364185.

### Functional predictions of the ASF community.

Using the annotated genomes, we predicted the metabolic potential of the ASF-defined microbiome by assigning KEGG orthology (KO) identifiers. As anticipated, the metabolic potential of the ASF covers a limited number of KO functions found in CONV animals. The ASF was predicted to cover 48% of KEGG modules, while other defined consortia of 15 (GM15) (47) or 12 (oligo-MM12) (23) cover 72 and 54% of modules, respectively (47). From another perspective, on average, each ASF member contributes a higher proportion toward KEGG module functions than more complex defined microbiota models. To identify the metabolic functions of the ASF, which represents the most “minimal” microbiome of the defined communities commonly reported, we compared the genetic content of the ASF with predicted functions encoded within the microbiota of CONV mice (Data Set S1 and S2). This resource can be used in a reductionistic approach to interrogate the microbiome by identifying functions that are or are not represented within the ASF community. This approach was successfully used to identify bacterial species that confer colonization resistance against Salmonella enterica serovar Typhimurium infection (23, 48) and to engineer the microbiome to treat hyperammonemia in mice (48). ASF-colonized mice have also been used to assess the function of microorganisms not found in the ASF, including the immunomodulatory properties of Bacteroides ovatus (49), fitness advantages of ADP-ribosyltransferase in Bacteroides stercoris (50), colonization properties of Oxalobacter formigenes (51) and the role of an Escherichia coli pathobiont in inducing inflammation (52). M. schaedleri (ASF457), a member of the poorly characterized phylum *Deferribacteres*, has also been characterized on its own to reveal insights into its interesting immunomodulatory properties (38) and ability to protect against bacterial infection (53).

10.1128/msystems.00293-22.6 DATA SET S1 ASF KEGG assignments. Download Data Set S1, XLSX file, 2.4 MB. Copyright © 2022 Proctor et al. 2022 Proctor et al. https://creativecommons.org/licenses/by/4.0/ This content is distributed under the terms of the Creative Commons Attribution 4.0 International license.

10.1128/msystems.00293-22.7 DATA SET S2 Heatmap of functional comparisons. Download Data Set S2, XLSX file, 0.02 MB. Copyright © 2022 Proctor et al. 2022 Proctor et al. https://creativecommons.org/licenses/by/4.0/ This content is distributed under the terms of the Creative Commons Attribution 4.0 International license.

**FIG 1:**
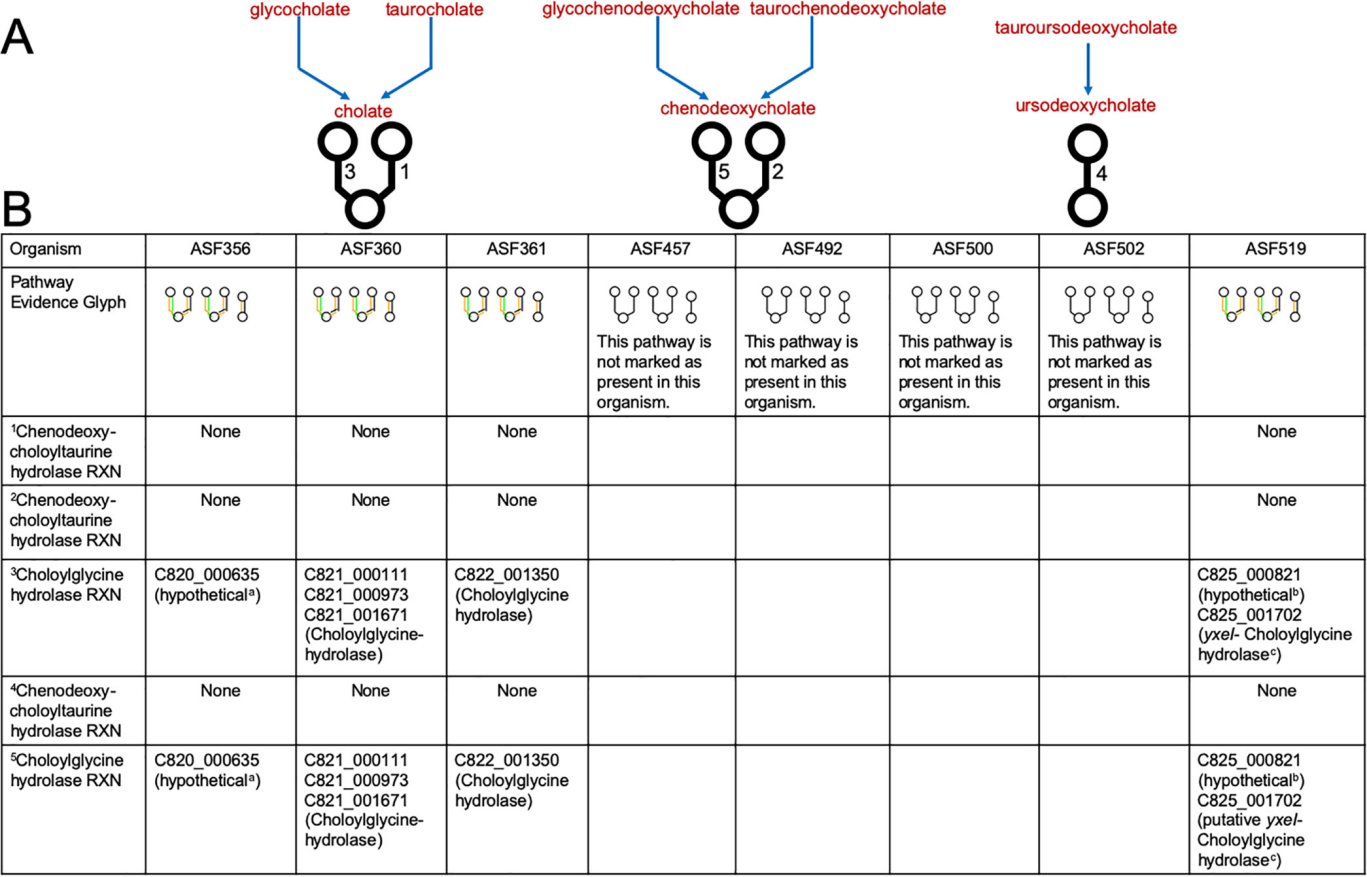
Identification and comparison of bile acid deconjugation pathways among ASF members by PTools software. (A) Five known pathways for metabolism of primary bile acids by bacterial deconjugation reactions. Specific deconjugation pathways are indicated by the glyphs below each group of conversions reactions. The numbers correspond to the specific enzymatic reactions (RXN) shown in the table. (B) The table shows the presence or absence of bile acid deconjugation pathways within each ASF member and was modified from the output of PTools. Green lines in the glyphs indicate that the pathway is present, while black lines indicate the pathway is not found. Orange lines indicate that reaction is unique to bile acid deconjugation compared to all other pathways in BioCyc. Inspection of the table revealed that choloylglycine hydrolase activity was predicted in ASF356, 360, 361, and 519, which was specific for the primary bile acids in pathways 3 and 5. No evidence of chenodeoxycholyltaurine hydrolase activity (pathways 1, 2, and 4) was found. In addition, no evidence of bile acid deconjugation activity was found in the genomes of ASF457, 492, 500, and 502. For the remaining ASF members, the locus tag and name of the enzymes they encode are shown. For the gene products listed as hypothetical or putative, the protein sequence was used to identify homologous proteins using BLASTp (NCBI) and including ^a^ASF356 (locus C820_000635), identical to choloylglycine hydrolase in *Clostridium* sp. Isolate MD294; ^b^ASF519 (locus C825_000821), identical to a linear amide C-N hydrolase in another Parabacteroides goldsteinii isolate; ^c^ASF519 (*yxeI*, locus C825_001702), highly similar to a choloylglycine hydrolase from Bacillus mohavensis.

To allow broad access to tools to analyze the ASF, we have taken advantage of recent developments on https://www.BioCyc.org (54) and the downloadable Pathway Tools (PTools) software (55, 56). Both environments support genome informatics (e.g., genome browser, sequence searching, multiple sequence alignment), pathway informatics (e.g., metabolic reconstruction, visualization of individual pathways and complete metabolic networks), comparative analysis (e.g., computation of orthologs and comparison of biosynthetic pathways across organisms) and ‘omics data analyses. New multiorganism search tools on https://www.BioCyc.org enable the user to define an organism set containing the ASF and search across the set for a given gene, gene product, metabolite, or pathway. To make this resource readily accessible to the scientific community, BioCyc Pathway/Genome Databases (PGDBs) were generated for each ASF member, which can now be found on https://www.BioCyc.org by selecting “Change Current Database” and searching for “ASF”. An example of the output of this analysis displaying the metabolic pathways ASF361 is shown in Fig. S1. We also made use of this tool to specifically compare bile acid metabolism among ASF members. Primary bile acids produced in the liver are metabolized by gut bacteria to generate derivatives that play important roles in lipid and carbohydrate metabolism, intestinal motility, and immune regulation (57). As shown in Fig. 1, ASF members are predicted to produce bile acid hydrolases (BSA) to deconjugate primary bile acids. Interestingly, ASF360, which primarily colonizes the GI tract proximal to the colon, encodes 3 distinct BSAs. In contrast, none of the ASF appear capable of 7α-dehydroxylation reactions, suggesting the ASF model may be useful to further study the role of specific secondary bile acids in host inflammatory responses (58). This easily accessed resource also represents a useful didactic tool for the microbiome using the ASF as a model system.

10.1128/msystems.00293-22.1 FIG S1 Functional predictions of ASF members from Pathway Tools analysis. Example of predicted metabolic pathways in ASF361 (Ligilactobacillus murinus). The names of pathways and metabolites are shown in small font as an example and can be identified using higher magnification by the Pathway Tools program. Download FIG S1, TIF file, 2.2 MB. Copyright © 2022 Proctor et al. 2022 Proctor et al. https://creativecommons.org/licenses/by/4.0/ This content is distributed under the terms of the Creative Commons Attribution 4.0 International license.

Inspection of the predicted metabolic pathways revealed insights into the functional capabilities of the ASF microbiome. Complete pathways for fundamental processes of microbial growth were observed, including central carbohydrate metabolism (e.g., glycolysis, pyruvate oxidation, pentose phosphate pathway), metabolism of glucuronate, galactose, glycogen, and nucleotide sugars, along with pathways for the biosynthesis of amino acids, nucleotides, vitamins, and other cofactors (Data Set S2). In contrast, several metabolic pathways found in the microbiota of CONV mice are completely missing in the ASF, which provides opportunities to explore their impact on the host. For example, pathways for xenobiotic biodegradation, including benzoate, anthranilate, catechol, and phenylacetate are either missing or incomplete in the ASF. The ASF also lacks the ability to synthesize specific antibiotics, such as puromycin, bacilysin, and fosfomycin, which could be useful to study microbial interactions within the gut. Likewise, the synthesis of neuro-active chemicals such as serotonin, melatonin, catecholamines, and kynurenine is limited. It has been shown ASF mice appear to be anxiolytic compared with CONV animals (59). Hence, the model offers a reductionistic system to study the gut-brain axis (60, 61). The functional limitations of the ASF provide the opportunity for investigators to introduce additional microorganisms to ASF mice to determine the impact of the expanded metabolic capabilities on the host and microbial community.

Within the ASF community, analysis of individual members using PTools allowed the identification of specific pathways encoded by each species. For example, ASF361 (Ligilactobacillus murinus), a predominant member of the ASF, encodes pathways to ferment a variety of sugars, including sucrose, galactose, mannose, fructose, maltose, melibiose, chitobiose, and *N*-acetylneuraminic acid, the latter of which is located on the terminal ends of mucins in the GI tract and may serve as a nutritional source (62, 63).

Loy et al. (64), reported the use of whole-genome sequencing to characterize ASF457, which included the identification of genes for flagellar biosynthesis, type six secretion systems (T6SS), and the ability to degrade glycans from the mucus layer. While biosynthetic pathways for homoserine, methionine, threonine, ornithine, and lysine were originally thought to be missing, genomic evidence was found that those pathways are complete or nearly complete in ASF457.

The role of horizontal gene transfer (HGT) is also highly relevant to microbiome function. Inspection of the annotated ASF519 genome reveals the presence of several mobile transposable elements. Given that P. goldsteinii is genetically tractable (65), it should be feasible to design experiments using the ASF to better understand the role of these elements in the gut.

### Quantification of the ASF members.

Because identifying changes in the abundance of bacterial species represents a fundamental approach to characterizing the microbiome, improved quantitative PCR (qPCR) assays were developed to measure the abundance of each ASF member. qPCR is an accurate method for quantitative microbiota profiling, including the determination of absolute abundance, as well as to improve the accuracy of sequencing-based methods (66, 67).

Primer pairs have been reported that targeted 16S rRNA genes of each of the ASF (27, 68). However, we sought a more robust qPCR assay for profiling the ASF community structure. For this, primer pairs were selected for the *groEL* (*cpn*) gene specific to each of the ASF (Table 2). *groEL* is a highly conserved gene found throughout *Eubacteria*, and sequence differences have been exploited for taxon-specific PCR primers (69–71). In contrast to 16S rRNA genes, targeting *groEL* simplifies the accurate determination of bacterial abundance as the gene is present in a single copy in the ASF members. Although the impact of different copy numbers on the accurate determination of microbial abundance continues to be debated (72–74), previous studies using qPCR were not able to account for differences in gene copy numbers within the ASF (27, 68, 75). While targeting the single copy *groEL* gene simplifies the interpretation of qPCR results, we reported that the copy numbers of 16S rRNA genes as: ASF356 (5); ASF360 (4); ASF361 (6); ASF457 (3); ASF492 (4); ASF500 (2); ASF502 (5); and ASF519 (6). These values can be used to reevaluate 16S rRNA gene qPCR or sequence data as necessary. The *groEL* primers (Table 2) were tested to ensure no cross-reactivity occurred among the ASF genomes. The primers also were designed to have nearly identical annealing temperatures to enable multiplexing reactions and specific amplicons can be generated without the need for nested PCRs (68).

**TABLE 2:** Quantitative and endpoint PCR primers

ASF number	Forward primer (5′–3′)	Reverse primer (5′–3′)	*T_m_*
qPCR primers			
356	GAT GGT GCT GGA AAC AAA GAA G	TAA CAG CAA CAC CAC CAG ATA A	56°C
360	TGG TGC TGC TAC TGA AA	CAC CAC CTG CAA CAT AAC	56°C
361	GCT GAA ACG ACC TCT GAA TTT G	GCG TCT TCG ATC CGG TAT TT	56°C
457	GAA GGA GCA GGT AAT ACT GAC G	TAG CAA GGC GTT CTT GAA GT	56°C
492	CAC GTC CTA ACT GCT CTA ATT C	GTC AAA GCT CCT GGT TAT GG	56°C
500	TGT CCA CCC TGA TTG TCA AC	ATC CTG AAG CAT CTC CTT GC	56°C
502	GGA TAC GGC GAC AGA AGA AA	CAT GGT GGT ATC CTT CAG ATC C	56°C
519	GGC GTT ATC ACA GTG GAA GA	GGT TAC GAA GTA CGG AGA GAT G	56°C
Endpoint primers^*a*^			
356	GGT ACT ATT AAT TGT GTT GCA GTG	CCA CCA GCA ACA ATA CCT T	62°C
360	GAT CAC TGA CAA GAA GAT TTC TAA C	CAC CAC CAG TTA ACT TAG CC	62°C
361	CCG TTA AAG CTC CAG GT	TAG CTA CAT CAT CGA TCA CAT C	62°C
457	AAA GCT CCT GGC TTT GG	GAG CTG AAA CAG TTC TTA CTA ATG	62°C
492	GCT GGA TCT TGT AGA AGG TAT G	TTC TTC GAT CTG GCT CTT AAT C	62°C
500	CCC TAT ATG GTC ACC GAC AC	TTG ATC TGG CCG ATG CG	62°C
502	GCG ACA GAT ATG GAG AAG ATG	CTC TTC GAT GCC CTT CTT G	62°C
519	TTA CAG CAG GTG CTA ACC	CAC GCA GAC GGT TTA CAA	62°C

a Each primer pair yielded an amplification product of ~500 bp in length.

**FIG 2:**
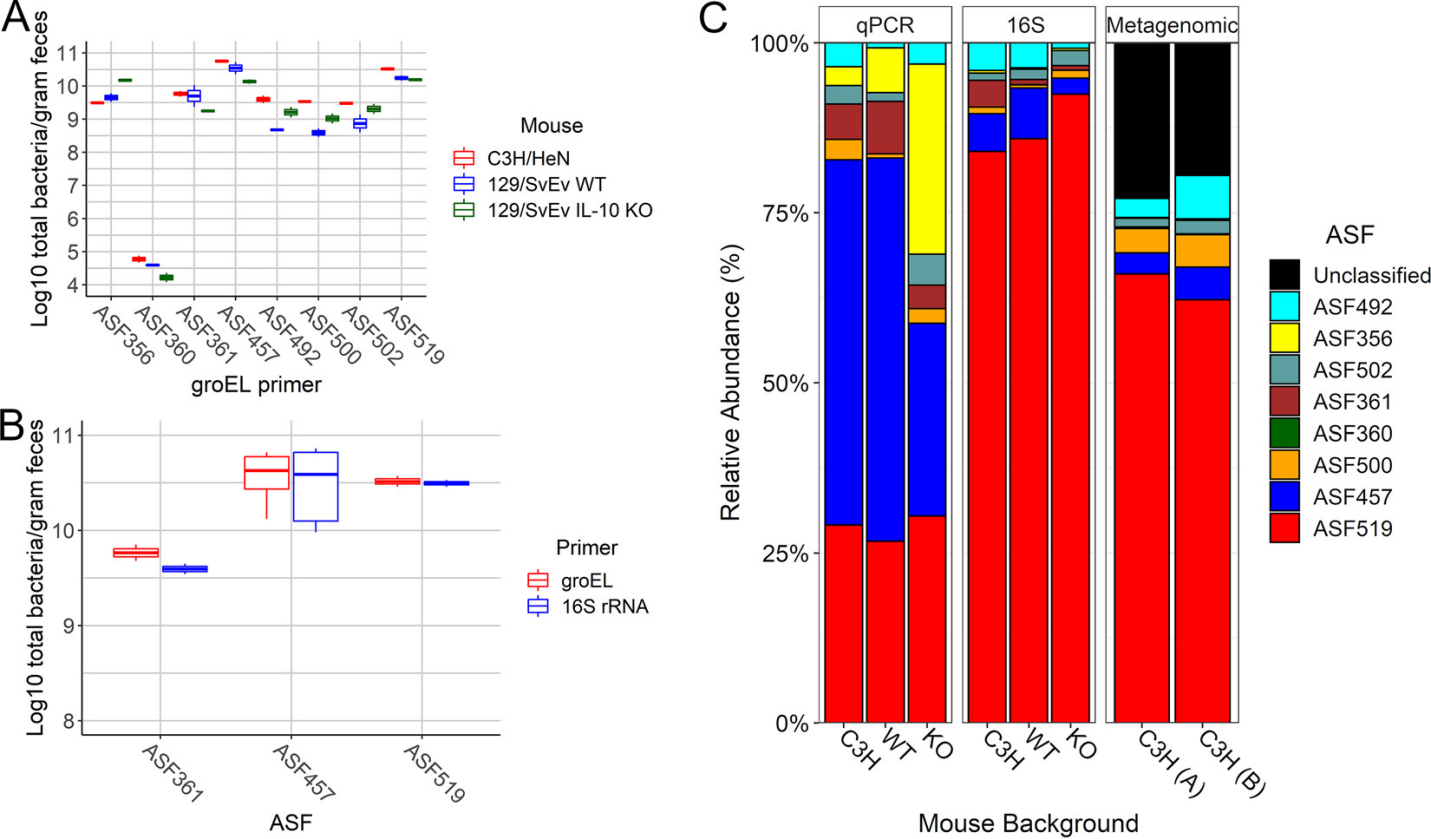
Abundance of the ASF in three strains of gnotobiotic mice. The absolute (A and B) and relative abundance (C) of the ASF were compared using different approaches. (A) The abundance of the ASF was compared between different mouse strains using *groEL* qPCR primers. (B) The abundance of ASF361, ASF457, and ASF519 was compared using *groEL* and 16S rRNA gene qPCR primers. (C) Relative abundance of the ASF using qPCR, 16S rRNA gene amplicon (16S) and metagenomic sequences from different mouse backgrounds: C3H/HeNTac (C3H) (*n* = 2 [qPCR], *n* = 13 [16S], *n* = 2 [metagenomics]); 129S6/SvEv wild type (WT) (*n* = 2 [qPCR], *n* = 7 [16S]) and 129S6/SvEv IL-10^−/−^ (KO) (*n* = 2 [qPCR], *n* = 5 [16S]).

To establish the relationship between gene copy number and Ct values, synthetic DNA constructs that include the regions targeted by each of the *groEL* qPCR primer pairs were designed (Table S1 and Fig. S2) and tested using DNA isolated from fecal samples to measure the abundance of each ASF member. Consistent with previous studies, the ASF members are not equally represented in the mouse gut (27, 68, 75), with ASF457 and ASF519 being the most predominant species in fecal samples (Fig. 2A). ASF356 and 361 were also found at high levels, with the remaining members found in nearly equal proportions. A low abundance of ASF360 in the fecal samples was also observed, which is consistent with the primary localization of the strain in the upper GI tract (27) and whose abundance is highly influenced by diet (76, 77). The results of *groEL* were also compared with ASF-specific 16S rRNA gene qPCR primers (68) for the most abundant ASF members. Fig. 2B shows the results are similar between the two primer sets for ASF361, 457, and 519.

10.1128/msystems.00293-22.2 FIG S2 Standard curves using *groEL* gBlocks. Standard curves for each ASF-specific synthetic DNA construct were prepared as described in Materials and Methods. Download FIG S2, TIF file, 2.5 MB. Copyright © 2022 Proctor et al. 2022 Proctor et al. https://creativecommons.org/licenses/by/4.0/ This content is distributed under the terms of the Creative Commons Attribution 4.0 International license.

10.1128/msystems.00293-22.4 TABLE S1 *groEL* gBlock sequences. Download Table S1, DOCX file, 0.02 MB. Copyright © 2022 Proctor et al. 2022 Proctor et al. https://creativecommons.org/licenses/by/4.0/ This content is distributed under the terms of the Creative Commons Attribution 4.0 International license.

It can also be desirable to qualitatively assess the presence or absence of specific ASF members or to confirm the identity of species after culturing *in vitro*. Hence, we also designed endpoint PCR primer pairs, each of which yields ~500-bp amplicon products (Table 2).

Other sequence-based approaches have been used to measure the abundance of ASF and comparisons of the results revealed abundance measurements, at least for contents of the distal colon and feces, vary with the methods used. For example, while ASF519 was the dominant member by 16S rRNA gene amplicon sequencing (51, 78) and metagenomic sequencing (48), 16S rRNA gene qPCR revealed that ASF457 is also highly abundant (27, 52, 75).

Because the microbiota can be influenced by diet and other rearing conditions (68, 75), we took advantage of ASF breeding colonies that included C3H/HeNTac mice, along with 129S6/SvEv and 129S6/SvEv IL-10^−/−^ isogenic mice to better understand how different methods to influence abundance measurements of the defined microbiota. IL-10 is a well-characterized immunomodulatory cytokine, and IL-10 knockout (IL-10^−/−^) mice are known to exhibit an altered microbiota (79–81). Abundance was measured using *groEL* qPCR, 16S rRNA gene amplicon sequencing and metagenomic sequencing using DNA isolated from fecal samples from individual ASF mice maintained under gnotobiotic conditions for over 5 years.

As shown in Fig. 2, qPCR revealed differences in absolute abundance between mouse strains, but none was statistically significant. However, while the relative abundance of the ASF was highly similar in C3H/HeNTac mice and wild-type 129S6/SvEv mice, the IL-10^−/−^ mice showed an increased abundance of ASF356 (Fig. 2C) indicating that the absence of the cytokine had a specific effect on the composition of the defined microbiota. Interestingly, while CONV IL-10^−/−^ mice develop spontaneous inflammation with age (82), ASF mice require the introduction of bacterial “provocateurs,” such as Helicobacter bilis, to trigger inflammation (32, 83).

Consistent with previous results from the ASF-colonized mice, the relative abundance measured by the two sequencing methods showed distinct differences with results obtained using qPCR (Fig. 2C). In particular, the levels of ASF519 were significantly higher, while ASF457 was substantially reduced with amplicon and metagenomic sequencing. These results reinforce previous observations that measurements of microbial abundance by DNA sequencing vary by the method used (84–86) and indicate the need for caution when interpreting results from different studies. Nonetheless, given the striking differences in abundance observed with ASF457 between qPCR and sequencing methods, we tested 6 additional primer pairs to target single copy genes unique to this strain (*hcpC*, *vasK*, *tssB*, *hylD*, *figK*, and a gene encoding a type I restriction-modification system component). In all cases, the Ct values of the qPCRs, and resulting abundance measurements, matched that obtained using *groEL* (and 16S rRNA gene) (unpublished data), further validating the use of the new primers and indicating that ASF457 is one of the most abundant members of the ASF consortium in the mice maintained in our gnotobiotic facility.

Given this, we routinely use *groEL* qPCR as a convenient, rapid, efficient, and cost-effective method to characterize and monitor the ASF community. In addition to the importance of measuring how the gut environment impacts bacterial abundance, there is also an interest in understanding the significance of the spatial distribution of microorganisms along the length of the gastrointestinal tract (27, 75). The ASF model, in combination with the resources reported here, should be a useful system to answer questions of microbiome “biogeography”. Furthermore, the ASF can provide a simplified and controlled bacterial community to explore other topics of gut microbial ecology, including analysis of community structure and host outcomes following colonization with fungi, protists, or bacteriophages (87).

### Global transcriptional analysis of the ASF.

Assessing transcriptional activity is a valuable approach to understanding how the microbiome responds to changes in the gut (88). Global transcriptome sequencing of the ASF has been used to develop data analysis pipelines (89, 90) and was recently employed to identify microbial genes that may contribute to the overall fitness and immunogenicity of the ASF (78). To further develop this method to understand microbiome function, we conducted a global transcriptome analysis to determine the extent to which treatments driving colonic inflammation and carcinogenesis influenced the structure and function of the defined ASF microbiome.

**FIG 3:**
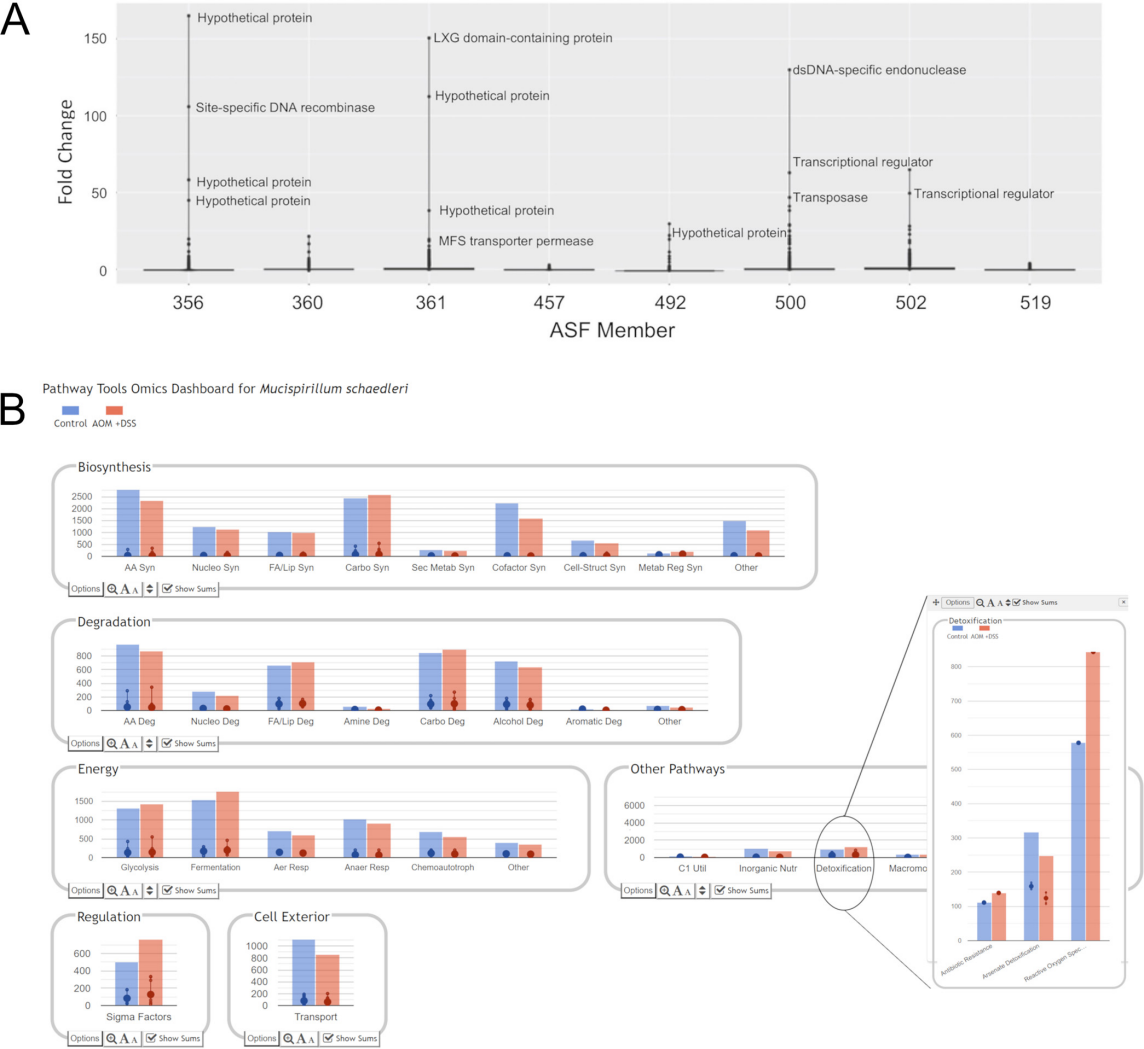
Treatment of mice with azoxymethane (AOM) plus dextran sulfate sodium (DSS) results in gene expression changes in the ASF. (A) Genes with the highest positive fold change were found in individual ASF members. (B) An example of the Pathway Tools ‘Omics Dashboard output used to assess the impact of AOM+DSS exposure on cellular functions of M. schaedleri (ASF457). Insert compared pathways involved in detoxification processes at higher resolution. Similar comparisons can be conducted on each ASF member using Pathway Tools. The ‘Omics Dashboard also allowed for more detailed visualization using the associated toolboxes at the bottom of each section.

As described in Materials and Methods, a well-established treatment regimen was used to expose mice to azoxymethane and dextran sodium sulfate (AOM+DSS) to induce inflammation that can lead to colonic tumors (91, 92). Although it has been reported that CONV C3H/HeNTac mice are not highly susceptible to the development of colonic tumors using AOM+DSS (93) and tumors were not detected following treatment of ASF C3H/HeNTac mice (data not shown), differential gene expression within the ASF community was observed following treatment (Data Set S3). While few genes showed differential expression in ASF457 and ASF519, the other members revealed differentially expressed genes. For this report, we identified genes whose expression increased greater than 20-fold following AOM+DSS treatment (Fig. 3A). These included genes that encode proteins that target DNA such as recombinases and restriction endonucleases (ASF356), an endonuclease (ASF500), transposases (ASF492 and ASF500) and genes encoding proteins of unknown function (hypothetical proteins). Transcriptional regulators were also identified in ASF360, ASF500, and ASF502, and the expression of the stress-induced chaperonin gene *groEL* increased in ASF360. In general, genes with increased expression were associated with DNA binding and repair suggesting a global stress response to AOM+DSS.

10.1128/msystems.00293-22.8 DATA SET S3 ASF RNAseq data. Download Data Set S3, XLSX file, 13.6 MB. Copyright © 2022 Proctor et al. 2022 Proctor et al. https://creativecommons.org/licenses/by/4.0/ This content is distributed under the terms of the Creative Commons Attribution 4.0 International license.

To facilitate analysis of the gene expression data, PTools software was used to identify metabolic pathways most affected by changes in transcriptional activity. As shown in Fig. 3B, this analysis visually revealed gene products that may influence other members of the gut community or the host. The global transcriptome data also revealed the ASF members did not respond equally to treatment.

A 16S rRNA gene amplicon sequencing of colon contents of the mice was also used to determine how the treatment influenced the relative abundance of the ASF. Inspection of the relative abundance and beta diversity of the two groups revealed in the AOM+DSS treated group, ASF519 and ASF492 decreased while ASF361 increased and revealed a separation between the two groups with the controls clustering more closely than the AOM+DSS group (Fig. S3).

10.1128/msystems.00293-22.3 FIG S3 Distribution of changes in ASF abundance following AOM+DSS treatment. (A) The relative distribution of the ASF between the control (left) and treatment (AOM+DSS) groups. (B) Weighted Unifrac PCoA plots show differential clustering between control animals (*n* = 8) (blue circles) and mice treated with AOM/DSS (*n* = 19) (red circles). Download FIG S3, TIF file, 0.6 MB. Copyright © 2022 Proctor et al. 2022 Proctor et al. https://creativecommons.org/licenses/by/4.0/ This content is distributed under the terms of the Creative Commons Attribution 4.0 International license.

### Genetic adaptation of the microbiome.

While the diversity of microbiome function is driven by the variety of microbial species in the mammalian gut, the contribution of genetic changes that lead to the emergence of species variants is much less well understood (94–96). Environmental perturbations, such as dietary changes, xenobiotics, and antibiotics can apply selective pressures that influence the functional composition of the microbiome on relatively short time scales (94). Addressing fundamental questions about how mutation and selection influence the function of the microbiome is challenging, in part due to the difficulty of distinguishing between species and species variants within CONV animals. Furthermore, multiple genetic processes such as mutations, recombination, and horizontal gene transfer contribute to genetic variation within the gut microbiome (97).

The ASF mouse model provides a unique system to study microbiome evolution over time and under different host environments as the sequenced genomes were generated from the original murine isolates obtained from Taconic Biosciences (25). To test the potential of using the ASF to address questions of microbial adaptation in the gut, we compared the genomes of each strain with shotgun metagenomic sequences derived from C3H/HeNTac mice that had been colonized with the ASF for over 22 years. Given the generation time for laboratory mice is 9 to 11 weeks (98), we estimate the C3H/HeNTac ASF mice have been maintained for 104 to 127 generations.

**FIG 4:**
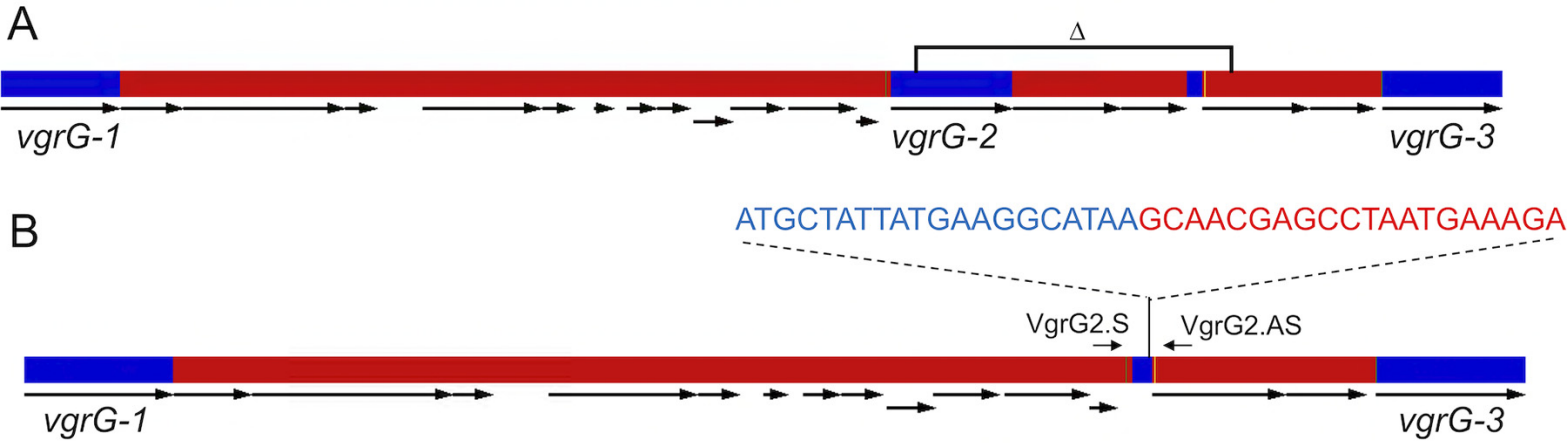
Genetic map of the M. schaedleri (ASF457) chromosome in the region encoding components of the type VI secretion system. (A) The location of the 3 homologous *vgrG* genes is shown, along with adjacent open reading frames (arrows). The location of the deletion mutation is shown by the square bracket. (B) The same region was identified from metagenomic sequences. The DNA sequence at the junction of the deletion is shown along with the location of PCR primers used to identify the deletion.

Sequence comparisons revealed numerous single nucleotide polymorphisms (SNPs), but little evidence of extensive insertions or deletions (indels) or HGT among the ASF was found (data not shown). We did observe, however, a genetic change in the T6SS machinery encoded by ASF457. As summarized in Fig. 4, the sequence of the M. schaedleri chromosome revealed 3 highly homologous copies of *vgrG* (Fig. 4A), which encodes the valine-glycine repeat protein and component of the T6SS spike protein complex (99). However, the metagenomic sequence of ASF457 showed that one of the 3 copies was deleted after becoming a member of the ASF consortium. This specific genetic change was confirmed by DNA sequencing of PCR amplicons targeting the *vgrG* region (Fig. 4B) and was observed in all C3H/HeNTac ASF mice tested. While the significance of this polymorphism is not yet known, we propose that the deletion of one of the copies of *vgrG* imparts a fitness advantage to ASF457 in the reduced complexity ASF microbiome. Given that ASF457, like the other members of the defined community, is commonly found in the microbiome of CONV mice (48, 75, 100), the ASF can be used to better understand how specific genetic adaptations determine microbiome function.

In conclusion, we have developed improved resources to facilitate the use of the minimalistic ASF mouse model to explore microbiome function and host-microbe interactions for systems-based approaches. The ASF is a well-established model that has been used for nearly 5 decades. The ASF consortium is highly stable, as evidenced by our maintenance of ASF mouse breeding colonies on different genetic backgrounds for as long as 22 years (24). Using standard methods of gnotobiotic husbandry and aseptic technique, GF mice of different genetic backgrounds can be colonized with the ASF. We have demonstrated that the resources reported here can be used to address questions of how changing the environment of the host’s gut through, for example, diet, drugs, or genetic background, impacts the structure and function of the ASF microbiome. The genetic content of the ASF has been incorporated into the BioCyc website and PTools software allowing convenient access to powerful tools to analyze the metabolic activity of the entire microbiome. In addition, a recent check indicates that live animal donors are available from Taconic Biosciences (Rensselaer, NY) to establish the ASF consortium in GF mice.

While the ASF community does not recapitulate the full functional capacity of the gut microbiota of CONV animals, this characteristic can be exploited by identifying specific metabolic pathways or functions missing in the ASF (Data Set S1 and S2) as a reductionistic approach to test the importance of the differences or an opportunity to add additional members to the community that carries the missing functions. Finally, we show that the ASF model can be used to better understand how bacterial genetic adaptation can influence the microbiome, which is challenging using conventional, more complex animal systems.

## MATERIALS AND METHODS

### *In vitro* culturing of the ASF.

Frozen stocks of each of the 8 ASF members (Table 1) were grown under anaerobic conditions (37°C consisting of 90% nitrogen, 5% carbon dioxide, and 5% hydrogen) in the 500-mL brain and heart infusion (BHI) broth supplemented with 5% (by volume) each of horse serum, sheep serum, and newborn calf serum for a total of 15% serum supplementation until turbid growth was achieved.

### Genomic DNA isolation, whole-genome sequencing, and annotation.

Total genomic DNA (gDNA) was isolated from 50 to 100 mL of pure cultures of each ASF strain grown from frozen stocks using Qiagen’s Genomic DNA buffer set and Qiagen QIAmp MidiPrep (100/G) columns using protocols described in the Qiagen Genomic DNA Handbook (08/2001) for isolating gDNA from bacteria. Additional lysozyme treatment was used before DNA extraction of ASF360 and ASF361. gDNA was suspended in 1× TE buffer and quantified using a Qubit 3.0 Fluorometer with the dsDNA BR assay kit (Life Technologies, Carlsbad, CA) before sequencing on the PacBio RSII platform using 1 to 2 SMRT cells per ASF and KAPA long insert library preparation (2 to 10 kb insertion) at the Institute for Genome Sciences, University of Maryland School of Medicine.

Sequences were assembled with HGAP (101) using reference ASF genomes from GenBank. Manual curation and refinement of assembled genomes were performed on anvio v7. Genomes were annotated using PROKKA (v1.14.16) (102). The predicted amino acid sequences from each annotated ASF genome were used as input for BlastKOALA (103) to assign KEGG Orthology terms. The updated genome annotations were submitted to BioCyc to create Pathway/Genome Databases (PGDBs) using the PathoLogic computational inference module of PathwayTools to infer metabolic pathways and operons.

### Animal studies and sample collection.

All animal protocols were conducted following the approval of ISU’s Institutional Animal Care and Use Committee. A colony of ASF mice on the C3H/HeNTac genetic background was originally obtained from Taconic Laboratories and has been maintained at ISU for over 22 years. The ASF was also established in 129S6/SvEv wild-type (WT) and IL-10 knockout (IL-10^−/−^) mice. Protocols for colonizing the ASF into GF mice are provided in Table S2.

10.1128/msystems.00293-22.5 TABLE S2 Colonization of the ASF into 129S6/SvEv mice. Download Table S2, DOCX file, 0.01 MB. Copyright © 2022 Proctor et al. 2022 Proctor et al. https://creativecommons.org/licenses/by/4.0/ This content is distributed under the terms of the Creative Commons Attribution 4.0 International license.

### PCR design and analysis.

The DNA sequences of the *groEL* gene of each ASF member were analyzed using PrimerQuest (Integrated DNA Technologies, Coralville, IA) to identify primers that distinguish between the ASF by both endpoint PCR and qPCR (Table 2). All qPCRs were performed in a QuantStudio 3 real-time PCR system (Applied Biosystems) using SYBR Green technology. Each reaction was prepared in triplicate in a 20-μL reaction volume using 1× Luna Universal qPCR Master Mix (New England Biolabs Inc.). qPCR was conducted using a program consisting of an initial denaturing step of 1 min at 95°C with 40 cycles at 56°C annealing for 15 s following 30 s of extension at 60°C. Melting curve analysis was included at the end of each program to verify the specificity of the amplification. Negative control reactions using nuclease-free water without DNA were generated at each run to ensure the purity of reagents.

Regions within each of the *groEL* genes amplified by the endpoint and qPCR primers were represented in ~500-bp synthetic gBlocks (IDT) (Table S1). The gBlocks were used to confirm that each primer pair was specific for its respective ASF member by checking for cross-reactivity between each primer pair. The 16S rRNA gene sequence qPCR primers employed in this study were published previously (27).

Standard curves for each ASF species were generated using a 10-fold serial dilution of *groEL*-specific gBlocks for standard curves, which ranged from 10 to 1010 copies. All calculations for generating curves were performed using a similar approach as described for ASF-specific primers based on the 16S rRNA gene (68). Primers to characterize the *vgrG* deletion mutants from ASF457 include VgrG2.S (5′-CCACAATGTATAGTAACAGGTGT-3′) and VgrG2.AS (5′-CGGTTCATTAGTCATATGGTCTT-3′). The resulting 450-bp PCR product was cloned in the vector pMini-T 2.0 (New England Biolabs), and the product was sequenced using plasmid-specific primers.

### AOM+DSS study.

Six- to eight-week-old immunocompetent ASF C3H/HeNTac mice were housed in germ-free conditions in HEPA-filtered cages for 10 weeks. AOM-treated mice were given a single intraperitoneal injection of azoxymethane (AOM, Midwest Research Institute) at a dose of 10 mg/kg body weight in week 1, as previously described (91). Control mice were given a single intraperitoneal injection of sterile saline at the same time. DSS (2% wt/vol) was added to the drinking water for 7 days with 2-week restitution between treatments for a total of 3 rounds of DSS exposure. After 10 weeks, mice were euthanized, and cecal and colon contents, as well as fecal pellets, were collected. The proximal 1 cm of the colon was collected in 10% formalin for a histopathology examination. Fecal pellets were stored at −80°C in RNALater Stabilization Solution (Thermo Fisher Scientific) for bacterial transcriptome analysis.

### RNA isolation and sequencing.

Total RNA was extracted from fecal pellets from ASF C3H/HeNTac mice after the final DSS treatment from the control and AOM+DSS treated mice using the MoBio PowerMicrobiome RNA isolation kit. RNA was pooled by the treatment group and quantified using the Qubit 2.0 Fluorometer with the Broad Range RNA Quantification kit. RNA was submitted to GENEWIZ (South Plainfield, NJ) for cDNA library preparation and sequencing on the Illumina HiSeq2500 platform using a 2 × 100-bp configuration.

Illumina Tru-Seq adaptors were trimmed using cutadapt (104) and sequences shorter than 50 bp were removed and quality filtering steps were performed using PRINSEQ with a minimum Phred score cutoff of 30 (105). Short reads from filtered metatranscriptomes were mapped onto each assembled ASF genome sequence to obtain mRNA expression and functional profiles. Transcripts were annotated using the database of Clusters of Orthologous Groups (COG) of proteins (106). Differential expression was determined using DeSeq2 (107).

### Genomic DNA Isolation from colon contents and feces.

The gDNA isolation was performed using the Qiagen DNeasy PowerSoil isolation kit on colon contents collected at necropsy using the manufacturer’s protocol for centrifugation. The purified gDNA was quantified, as described above, and stored at −20°C in the supplied 10 mM Tris Buffer.

### 16S rRNA gene amplicon sequencing.

The gDNA isolated from colon contents (AOM+DSS study) or feces (all others) was used as the template for PCR amplification of the V4 variable region of the 16S rRNA gene sequence with specific primers (515f-816r). Amplicon sequencing was performed by the Institute for Genomics and Systems Biology at the Argonne National Laboratory (Argonne, IL) on the Illumina MiSeq Platform. Sequences were analyzed using QIIME 1.9 (Quantitative Insights into Microbial Ecology) (108) as previously described (31).

### Metagenomic sequencing.

The gDNA was isolated from colon contents of two six-week-old ASF-C3H/HeNTac mice (samples C3H/HeN-Shotgun Metagenomic-A and C3H/HeN-Shotgun Metagenomic-B) and sequenced (BGI, Cambridge, MA) on the Illumina HiSeq 4000 using 150-bp paired-end sequencing. A total of 52,042,370 reads for C3H/HeN-Shotgun Metagenomic-A and 48,741,166 reads for C3H/HeN-Shotgun Metagenomic-B were obtained, which were mapped to individual ASF genomes using BWA (v0.7.13) (109). Sequences with a Phred score <20, greater than 10% N’s, or adapter contamination were removed before genome assembly. The genome size and degrees of duplication and heterozygosity for each sample were estimated using k-mer analysis. GC estimation was performed after genome assembly using GC-Depth analysis. The relative abundance of the ASF was calculated based on sequence mapping to each ASF genome.

### Data availability.

Genome sequences of the ASF are available at GenBank with the accession numbers shown in Table 1. Results of 16S rRNA gene amplicon sequencing were deposited in GenBank under accession number PRJNA844334. Metatranscriptomic and 16S rRNA gene amplicon sequencing associated with the AOM+DSS colitis model was deposited under BioProject number PRJNA772742. Metagenomic sequencing results were deposited under BioProject number PRJNA838940. Protein/Genome Data Base (PGDB) files of each ASF strain can be found by going to https://www.biocyc.org, selecting “Change Current Database,” and searching the ASF member in the bar under “Select a Database”. Sequences of endpoint and qPCR primers are shown in Table 2 and the sequences of *groEL* gBlocks are shown in Table S2.

## References

[1] Lynch SV, Pedersen O. 2016. The human intestinal microbiome in health and disease. N Engl J Med 375:2369–2379. doi:10.1056/NEJMra1600266. 27974040

[2] Rooks MG, Garrett WS. 2016. Gut microbiota, metabolites and host immunity. Nat Rev Immunol 16:341–352. doi:10.1038/nri.2016.42. 27231050 PMC5541232

[3] Schmidt TSB, Raes J, Bork P. 2018. The human gut microbiome: from association to modulation. Cell 172:1198–1215. doi:10.1016/j.cell.2018.02.044. 29522742

[4] Vuong HE, Yano JM, Fung TC, Hsiao EY. 2017. The microbiome and host behavior. Annu Rev Neurosci 40:21–49. doi:10.1146/annurev-neuro-072116-031347. 28301775 PMC6661159

[5] Human Microbiome Project Consortium. 2012. Structure, function and diversity of the healthy human microbiome. Nature 486:207–214. doi:10.1038/nature11234. 22699609 PMC3564958

[6] Wang J, Lang T, Shen J, Dai J, Tian L, Wang X. 2019. Core gut bacteria analysis of healthy mice. Front Microbiol 10:887. doi:10.3389/fmicb.2019.00887. 31105675 PMC6491893

[7] Yeoman CJ, Chia N, Jeraldo P, Sipos M, Goldenfeld ND, White BA. 2012. The microbiome of the chicken gastrointestinal tract. Anim Health Res Rev 13:89–99. doi:10.1017/S1466252312000138. 22853945

[8] Tierney BT, Yang Z, Luber JM, Beaudin M, Wibowo MC, Baek C, Mehlenbacher E, Patel CJ, Kostic AD. 2019. The landscape of genetic content in the gut and oral human microbiome. Cell Host Microbe 26:283–295.e8. doi:10.1016/j.chom.2019.07.008. 31415755 PMC6716383

[9] Zhu J, Ren H, Zhong H, Li X, Zou Y, Han M, Li M, Madsen L, Kristiansen K, Xiao L. 2021. An expanded gene catalog of mouse gut metagenomes. mSphere 6:e01119-20. doi:10.1128/mSphere.01119-20. 33627510 PMC8544893

[10] Douglas AE. 2019. Simple animal models for microbiome research. Nat Rev Microbiol 17:764–775. doi:10.1038/s41579-019-0242-1. 31417197

[11] Kim BS, Kim JN, Cerniglia CE. 2011. *In vitro* culture conditions for maintaining a complex population of human gastrointestinal tract microbiota. J Biomed Biotechnol 2011:838040. doi:10.1155/2011/838040. 21811382 PMC3147118

[12] Krych L, Hansen CH, Hansen AK, van den Berg FW, Nielsen DS. 2013. Quantitatively different, yet qualitatively alike: a meta-analysis of the mouse core gut microbiome with a view towards the human gut microbiome. PLoS One 8:e62578. doi:10.1371/journal.pone.0062578. 23658749 PMC3641060

[13] Nguyen TL, Vieira-Silva S, Liston A, Raes J. 2015. How informative is the mouse for human gut microbiota research? Dis Model Mech 8:1–16. doi:10.1242/dmm.017400. 25561744 PMC4283646

[14] Ericsson AC, Franklin CL. 2015. Manipulating the gut microbiota: methods and challenges. Ilar J 56:205–217. doi:10.1093/ilar/ilv021. 26323630 PMC4554251

[15] Grover M, Kashyap PC. 2014. Germ-free mice as a model to study effect of gut microbiota on host physiology. Neurogastroenterol Motil 26:745–748. doi:10.1111/nmo.12366. 24860967 PMC4083815

[16] Kostopoulos I, Aalvink S, Kovatcheva-Datchary P, Nijsse B, Bäckhed F, Knol J, de Vos WM, Belzer C. 2021. A continuous battle for host-derived glycans between a mucus specialist and a glycan generalist *in vitro* and *in vivo*. Front Microbiol 12:632454. doi:10.3389/fmicb.2021.632454. 34248864 PMC8264420

[17] Cuenca M, Pfister SP, Buschor S, Bayramova F, Hernandez SB, Cava F, Kuru E, Van Nieuwenhze MS, Brun YV, Coelho FM, Hapfelmeier S. 2016. D-alanine-controlled transient intestinal mono-colonization with non-laboratory-adapted commensal *E. coli* strain HS. PLoS One 11:e0151872. doi:10.1371/journal.pone.0151872. 27002976 PMC4803232

[18] Grimm V, Radulovic K, Riedel CU. 2015. Colonization of C57BL/6 mice by a potential probiotic *Bifidobacterium bifidum* strain under germ-free and specific pathogen-free conditions and during experimental colitis. PLoS One 10:e0139935. doi:10.1371/journal.pone.0139935. 26439388 PMC4595203

[19] Elzinga J, van der Oost J, de Vos WM, Smidt H. 2019. The use of defined microbial communities to model host-microbe interactions in the Human Gut. Microbiol Mol Biol Rev 83:1–40. doi:10.1128/MMBR.00054-18. PMC6684003 30867232

[20] Lee SM, Donaldson GP, Mikulski Z, Boyajian S, Ley K, Mazmanian SK. 2013. Bacterial colonization factors control specificity and stability of the gut microbiota. Nature 501:426–429. doi:10.1038/nature12447. 23955152 PMC3893107

[21] Lofgren JL, Whary MT, Ge Z, Muthupalani S, Taylor NS, Mobley M, Potter A, Varro A, Eibach D, Suerbaum S, Wang TC, Fox JG. 2011. Lack of commensal flora in *Helicobacter pylori*-infected INS-GAS mice reduces gastritis and delays intraepithelial neoplasia. Gastroenterology 140:210–220. doi:10.1053/j.gastro.2010.09.048. 20950613 PMC3006487

[22] Becker N, Kunath J, Loh G, Blaut M. 2011. Human intestinal microbiota: characterization of a simplified and stable gnotobiotic rat model. Gut Microbes 2:25–33. doi:10.4161/gmic.2.1.14651. 21637015

[23] Brugiroux S, Beutler M, Pfann C, Garzetti D, Ruscheweyh HJ, Ring D, Diehl M, Herp S, Lötscher Y, Hussain S, Bunk B, Pukall R, Huson DH, Münch PC, McHardy AC, McCoy KD, Macpherson AJ, Loy A, Clavel T, Berry D, Stecher B. 2016. Genome-guided design of a defined mouse microbiota that confers colonization resistance against *Salmonella enterica* serovar Typhimurium. Nat Microbiol 2:16215. doi:10.1038/nmicrobiol.2016.215. 27869789

[24] Wymore Brand M, Wannemuehler MJ, Phillips GJ, Proctor A, Overstreet AM, Jergens AE, Orcutt RP, Fox JG. 2015. The altered Schaedler flora: continued applications of a defined murine microbial community. Ilar J 56:169–178. doi:10.1093/ilar/ilv012. 26323627 PMC4554250

[25] Orcutt RP, Gianni FJ, Judge RJ. 1987. Development of an “altered Schaedler flora” for NCI gnotobiotic rodents. Microecology and Therapy 17:59.

[26] Dewhirst FE, Chien CC, Paster BJ, Ericson RL, Orcutt RP, Schauer DB, Fox JG. 1999. Phylogeny of the defined murine microbiota: altered Schaedler flora. Appl Environ Microbiol 65:3287–3292. doi:10.1128/AEM.65.8.3287-3292.1999. 10427008 PMC91493

[27] Sarma-Rupavtarm RB, Ge Z, Schauer DB, Fox JG, Polz MF. 2004. Spatial distribution and stability of the eight microbial species of the altered schaedler flora in the mouse gastrointestinal tract. Appl Environ Microbiol 70:2791–2800. doi:10.1128/AEM.70.5.2791-2800.2004. 15128534 PMC404395

[28] Stehr M, Greweling MC, Tischer S, Singh M, Blöcker H, Monner DA, Müller W. 2009. Charles River altered Schaedler flora (CRASF) remained stable for four years in a mouse colony housed in individually ventilated cages. Lab Anim 43:362–370. doi:10.1258/la.2009.0080075. 19535393

[29] Rooks MG, Veiga P, Reeves AZ, Lavoie S, Yasuda K, Asano Y, Yoshihara K, Michaud M, Wardwell-Scott L, Gallini CA, Glickman JN, Sudo N, Huttenhower C, Lesser CF, Garrett WS. 2017. QseC inhibition as an antivirulence approach for colitis-associated bacteria. Proc Natl Acad Sci USA 114:142–147. doi:10.1073/pnas.1612836114. 27980034 PMC5224399

[30] Stromberg ZR, Van Goor A, Redweik GAJ, Wymore Brand MJ, Wannemuehler MJ, Mellata M. 2018. Pathogenic and non-pathogenic *Escherichia coli* colonization and host inflammatory response in a defined microbiota mouse model. Dis Model Mech 11:dmm035063. doi:10.1242/dmm.035063. 30275104 PMC6262807

[31] Atherly T, Mosher C, Wang C, Hostetter J, Proctor A, Wymore-Brand M, Phillips GJ, Wannemuehler M, Jergens AE. 2016. *Helicobacter bilis* infection alters mucosal bacteria and modulates colitis development in defined microbiota mice. Inflamm Bowel Dis 22:2571–2581. doi:10.1097/MIB.0000000000000944. 27755267 PMC5123692

[32] Jergens AE, Dorn A, Wilson J, Dingbaum K, Henderson A, Liu Z, Hostetter J, Evans RB, Wannemuehler MJ. 2006. Induction of differential immune reactivity to members of the flora of gnotobiotic mice following colonization with *Helicobacter bilis* or *Brachyspira hyodysenteriae*. Microbes Infect 8:1602–1610. doi:10.1016/j.micinf.2006.01.019. 16698302

[33] Stecher B, Chaffron S, Käppeli R, Hapfelmeier S, Freedrich S, Weber TC, Kirundi J, Suar M, McCoy KD, von Mering C, Macpherson AJ, Hardt WD. 2010. Like will to like: abundances of closely related species can predict susceptibility to intestinal colonization by pathogenic and commensal bacteria. PLoS Pathog 6:e1000711. doi:10.1371/journal.ppat.1000711. 20062525 PMC2796170

[34] Bolsega S, Basic M, Smoczek A, Buettner M, Eberl C, Ahrens D, Odum KA, Stecher B, Bleich A. 2019. Composition of the intestinal microbiota determines the outcome of virus-triggered colitis in mice. Front Immunol 10:1708. doi:10.3389/fimmu.2019.01708. 31396223 PMC6664081

[35] Geuking MB, Cahenzli J, Lawson MA, Ng DC, Slack E, Hapfelmeier S, McCoy KD, Macpherson AJ. 2011. Intestinal bacterial colonization induces mutualistic regulatory T cell responses. Immunity 34:794–806. doi:10.1016/j.immuni.2011.03.021. 21596591

[36] Mosconi I, Geuking MB, Zaiss MM, Massacand JC, Aschwanden C, Kwong Chung CK, McCoy KD, Harris NL. 2013. Intestinal bacteria induce TSLP to promote mutualistic T-cell responses. Mucosal Immunol 6:1157–1167. doi:10.1038/mi.2013.12. 23515135

[37] Natividad JM, Petit V, Huang X, de Palma G, Jury J, Sanz Y, Philpott D, Garcia Rodenas CL, McCoy KD, Verdu EF. 2012. Commensal and probiotic bacteria influence intestinal barrier function and susceptibility to colitis in Nod1−/−; Nod2−/− mice. Inflamm Bowel Dis 18:1434–1446. doi:10.1002/ibd.22848. 22162005

[38] Campbell C, Dikiy S, Bhattarai SK, Chinen T, Matheis F, Calafiore M, Hoyos B, Hanash A, Mucida D, Bucci V, Rudensky AY. 2018. Extrathymically generated regulatory T cells establish a niche for intestinal border-dwelling bacteria and affect physiologic metabolite balance. Immunity 48:1245–1257.e9. doi:10.1016/j.immuni.2018.04.013. 29858010 PMC6260932

[39] Bayer F, Ascher S, Kiouptsi K, Kittner JM, Stauber RH, Reinhardt C. 2021. Colonization with altered Schaedler flora impacts leukocyte adhesion in mesenteric ischemia-reperfusion injury. Microorganisms 9:1601. doi:10.3390/microorganisms9081601. 34442681 PMC8401286

[40] Moghadamrad S, McCoy KD, Geuking MB, Sägesser H, Kirundi J, Macpherson AJ, De Gottardi A. 2015. Attenuated portal hypertension in germ-free mice: function of bacterial flora on the development of mesenteric lymphatic and blood vessels. Hepatology 61:1685–1695. doi:10.1002/hep.27698. 25643846

[41] Moghadamrad S, Hassan M, McCoy KD, Kirundi J, Kellmann P, De Gottardi A. 2019. Attenuated fibrosis in specific pathogen-free microbiota in experimental cholestasis- and toxin-induced liver injury. FASEB J 33:12464–12476. doi:10.1096/fj.201901113R. 31431085 PMC6902738

[42] Biggs MB, Medlock GL, Moutinho TJ, Lees HJ, Swann JR, Kolling GL, Papin JA. 2017. Systems-level metabolism of the altered Schaedler flora, a complete gut microbiota. ISME J 11:426–438. doi:10.1038/ismej.2016.130. 27824342 PMC5270571

[43] Medlock GL, Carey MA, McDuffie DG, Mundy MB, Giallourou N, Swann JR, Kolling GL, Papin JA. 2018. Inferring metabolic mechanisms of interaction within a defined gut microbiota. Cell Syst 7:245–257.e7. doi:10.1016/j.cels.2018.08.003. 30195437 PMC6166237

[44] Wannemuehler MJ, Overstreet AM, Ward DV, Phillips GJ. 2014. Draft genome sequences of the altered schaedler flora, a defined bacterial community from gnotobiotic mice. Genome Announc 2:e00287-14. doi:10.1128/genomeA.00287-14. 24723722 PMC3983311

[45] Meier-Kolthoff JP, Göker M. 2019. TYGS is an automated high-throughput platform for state-of-the-art genome-based taxonomy. Nat Commun 10:2182. doi:10.1038/s41467-019-10210-3. 31097708 PMC6522516

[46] Meier-Kolthoff JP, Carbasse JS, Peinado-Olarte RL, Göker M. 2022. TYGS and LPSN: a database tandem for fast and reliable genome-based classification and nomenclature of prokaryotes. Nucleic Acids Res 50:D801–D807. doi:10.1093/nar/gkab902. 34634793 PMC8728197

[47] Darnaud M, De Vadder F, Bogeat P, Boucinha L, Bulteau A-L, Bunescu A, Couturier C, Delgado A, Dugua H, Elie C, Mathieu A, Novotná T, Ouattara DA, Planel S, Saliou A, Šrůtková D, Yansouni J, Stecher B, Schwarzer M, Leulier F, Tamellini A. 2021. A standardized gnotobiotic mouse model harboring a minimal 15-member mouse gut microbiota recapitulates SOPF/SPF phenotypes. Nat Commun 12:6686. doi:10.1038/s41467-021-26963-9. 34795236 PMC8602333

[48] Shen TC, Albenberg L, Bittinger K, Chehoud C, Chen YY, Judge CA, Chau L, Ni J, Sheng M, Lin A, Wilkins BJ, Buza EL, Lewis JD, Daikhin Y, Nissim I, Yudkoff M, Bushman FD, Wu GD. 2015. Engineering the gut microbiota to treat hyperammonemia. J Clin Invest 125:2841–2850. doi:10.1172/JCI79214. 26098218 PMC4563680

[49] Lavoie S, Conway KL, Lassen KG, Jijon HB, Pan H, Chun E, Michaud M, Lang JK, Gallini Comeau CA, Dreyfuss JM, Glickman JN, Vlamakis H, Ananthakrishnan A, Kostic A, Garrett WS, Xavier RJ. 2019. The Crohn's disease polymorphism, *Atg16l1* t300a, alters the gut microbiota and enhances the local Th1/Th17 response. Elife 8:e39982. doi:10.7554/eLife.39982. 30666959 PMC6342529

[50] Brown K, Zaytsoff SJ, Uwiera RR, Inglis GD. 2016. Antimicrobial growth promoters modulate host responses in mice with a defined intestinal microbiota. Sci Rep 6:38377. doi:10.1038/srep38377. 27929072 PMC5144068

[51] Li X, Ellis ML, Dowell AE, Kumar R, Morrow CD, Schoeb TR, Knight J. 2016. Response of germ-free mice to colonization with *O. formigenes* and altered schaedler flora. Appl Environ Microbiol 82:6952–6960. doi:10.1128/AEM.02381-16. 27663026 PMC5103094

[52] Chassaing B, Gewirtz AT. 2018. Mice harboring pathobiont-free microbiota do not develop intestinal inflammation that normally results from an innate immune deficiency. PLoS One 13:e0195310. doi:10.1371/journal.pone.0195310. 29617463 PMC5884553

[53] Herp S, Brugiroux S, Garzetti D, Ring D, Jochum LM, Beutler M, Eberl C, Hussain S, Walter S, Gerlach RG, Ruscheweyh HJ, Huson D, Sellin ME, Slack E, Hanson B, Loy A, Baines JF, Rausch P, Basic M, Bleich A, Berry D, Stecher B. 2019. *Mucispirillum schaedleri* antagonizes *Salmonella* virulence to protect mice against colitis. Cell Host Microbe 25:681–694.e8. doi:10.1016/j.chom.2019.03.004. 31006637

[54] Paley S, Karp PD. 2021. The BioCyc metabolic network explorer. BMC Bioinformatics 22:208. doi:10.1186/s12859-021-04132-5. 33882841 PMC8060992

[55] Karp PD, Midford PE, Billington R, Kothari A, Krummenacker M, Latendresse M, Ong WK, Subhraveti P, Caspi R, Fulcher C, Keseler IM, Paley SM. 2021. Pathway Tools version 23.0 update: software for pathway/genome informatics and systems biology. Brief Bioinform 22:109–126. doi:10.1093/bib/bbz104. 31813964 PMC8453236

[56] Karp PD, Midford PE, Paley SM, Krummenacker M, Billington R, Kothari A, Ong WK, Subhraveti P, Keseler IM, Caspi R. 2019. Pathway Tools version 23.0: integrated software for pathway/genome informatics and systems biology. arXiv:1–111. doi:10.48550/arXiv.1510.03964. PMC8453236 31813964

[57] Joyce SA, Gahan CGM. 2017. Disease-associated changes in bile acid profiles and links to altered gut microbiota. Dig Dis 35:169–177. doi:10.1159/000450907. 28249284

[58] Duboc H, Rajca S, Rainteau D, Benarous D, Maubert M-A, Quervain E, Thomas G, Barbu V, Humbert L, Despras G, Bridonneau C, Dumetz F, Grill J-P, Masliah J, Beaugerie L, Cosnes J, Chazouillères O, Poupon R, Wolf C, Mallet J-M, Langella P, Trugnan G, Sokol H, Seksik P. 2013. Connecting dysbiosis, bile-acid dysmetabolism and gut inflammation in inflammatory bowel diseases. Gut 62:531–539. doi:10.1136/gutjnl-2012-302578. 22993202

[59] Lyte JM, Proctor A, Phillips GJ, Lyte M, Wannemuehler M. 2019. Altered Schaedler flora mice: a defined microbiota animal model to study the microbiota-gut-brain axis. Behav Brain Res 356:221–226. doi:10.1016/j.bbr.2018.08.022. 30153465

[60] Morais LH, Schreiber H, Mazmanian SK. 2021. The gut microbiota-brain axis in behaviour and brain disorders. Nat Rev Microbiol 19:241–255. doi:10.1038/s41579-020-00460-0. 33093662

[61] Valles-Colomer M, Falony G, Darzi Y, Tigchelaar ET, Wang J, Tito RY, Schiweck C, Kurilshikov A, Joossens M, Wijmenga C, Claes S, Van Oudenhove L, Zhernakova A, Vieira-Silva S, Raes J. 2019. The neuroactive potential of the human gut microbiota in quality of life and depression. Nat Microbiol 4:623–632. doi:10.1038/s41564-018-0337-x. 30718848

[62] Juge N, Tailford L, Owen CD. 2016. Sialidases from gut bacteria: a mini-review. Biochem Soc Trans 44:166–175. doi:10.1042/BST20150226. 26862202 PMC4747158

[63] Larsson JMH, Thomsson KA, Rodríguez-Piñeiro AM, Karlsson H, Hansson GC. 2013. Studies of mucus in mouse stomach, small intestine, and colon. III. Gastrointestinal Muc5ac and Muc2 mucin O-glycan patterns reveal a regiospecific distribution. Am J Physiol Gastrointest Liver Physiol 305:357–363. doi:10.1152/ajpgi.00048.2013. PMC3761246 23832516

[64] Loy A, Pfann C, Steinberger M, Hanson B, Herp S, Brugiroux S, Gomes Neto JC, Boekschoten MV, Schwab C, Urich T, Ramer-Tait AE, Rattei T, Stecher B, Berry D. 2017. Lifestyle and horizontal gene transfer-mediated evolution of *Mucispirillum schaedleri*, a core member of the murine gut microbiota. mSystems 2:e00171-16. doi:10.1128/mSystems.00171-16. 28168224 PMC5285517

[65] García-Bayona L, Comstock LE. 2019. Streamlined genetic manipulation of diverse bacteroides and parabacteroides isolates from the human gut microbiota. mBio 10:e01762-19. doi:10.1128/mBio.01762-19. 31409684 PMC6692515

[66] Dannemiller KC, Lang-Yona N, Yamamoto N, Rudich Y, Peccia J. 2014. Combining real-time PCR and next generation DNA sequencing to provide quantitative comparisons of fungal aerosol populations. Atmospheric Environment 84:113–121. doi:10.1016/j.atmosenv.2013.11.036.

[67] Jian C, Luukkonen P, Yki-Järvinen H, Salonen A, Korpela K. 2020. Quantitative PCR provides a simple and accessible method for quantitative microbiota profiling. PLoS One 15:e0227285. doi:10.1371/journal.pone.0227285. 31940382 PMC6961887

[68] Gomes-Neto JC, Mantz S, Held K, Sinha R, Segura Munoz RR, Schmaltz R, Benson AK, Walter J, Ramer-Tait AE. 2017. A real-time PCR assay for accurate quantification of the individual members of the Altered Schaedler Flora microbiota in gnotobiotic mice. J Microbiol Methods 135:52–62. doi:10.1016/j.mimet.2017.02.003. 28189782 PMC5365401

[69] Chen Q, Wu G, Chen H, Li H, Li S, Zhang C, Pang X, Wang L, Zhao L, Shen J. 2019. Quantification of human oral and fecal *Streptococcus parasanguinis* by use of quantitative real-time PCR targeting the *groEL* gene. Front Microbiol 10:2910. doi:10.3389/fmicb.2019.02910. 31921079 PMC6933288

[70] Forghani F, Wei S, Oh DH. 2016. A rapid multiplex real-time PCR high-resolution melt curve assay for the simultaneous detection of *Bacillus cereus*, *Listeria monocytogenes*, and *Staphylococcus aureus* in food. J Food Prot 79:810–815. doi:10.4315/0362-028X.JFP-15-428. 27296430

[71] Junick J, Blaut M. 2012. Quantification of human fecal *Bifidobacterium* species by use of quantitative real-time PCR analysis targeting the *groEL* gene. Appl Environ Microbiol 78:2613–2622. doi:10.1128/AEM.07749-11. 22307308 PMC3318781

[72] Louca S, Doebeli M, Wegener Parfrey L. 2018. Correcting for 16s rrna gene copy numbers in microbiome surveys remains an unsolved problem. Microbiome 6:41. doi:10.1186/s40168-018-0420-9. 29482646 PMC5828423

[73] Bonk F, Popp D, Harms H, Centler F. 2018. Pcr-based quantification of taxa-specific abundances in microbial communities: quantifying and avoiding common pitfalls. J Microbiol Methods 153:139–147. doi:10.1016/j.mimet.2018.09.015. 30267718

[74] Starke R, Satler Pylro V, Kumazawa Morais D. 2021. 16s rrna gene copy number normalization does not provide more reliable conclusions in metataxonomic surveys. Microb Ecol 81:535–539. doi:10.1007/s00248-020-01586-7. 32862246 PMC7835310

[75] Alexander AD, Orcutt RP, Henry JC, Baker JJ, Bissahoyo AC, Threadgill DW. 2006. Quantitative PCR assays for mouse enteric flora reveal strain-dependent differences in composition that are influenced by the microenvironment. Mamm Genome 17:1093–1104. doi:10.1007/s00335-006-0063-1. 17091319

[76] Dubos R, Schaedler RW, Costello R, Hoet P. 1965. Indigenous, normal, and autochthonous flora of the gastrointestinal tract. J Exp Med 122:67–76. doi:10.1084/jem.122.1.67. 14325474 PMC2138034

[77] Dubos RJ, Schaedler RW. 1962. The effect of diet on the fecal bacterial flora of mice and on their resistance to infection. J Exp Med 115:1161–1172. doi:10.1084/jem.115.6.1161. 13888074 PMC2137396

[78] Tsou AM, Goettel JA, Bao B, Biswas A, Kang YH, Redhu NS, Peng K, Putzel GG, Saltzman J, Kelly R, Gringauz J, Barends J, Hatazaki M, Frei SM, Emani R, Huang Y, Shen Z, Fox JG, Glickman JN, Horwitz BH, Snapper SB. 2021. Utilizing a reductionist model to study host-microbe interactions in intestinal inflammation. Microbiome 9:215. doi:10.1186/s40168-021-01161-3. 34732258 PMC8565002

[79] Overstreet AC, Ramer-Tait AE, Suchodolski JS, Hostetter JM, Wang C, Jergens AE, Phillips GJ, Wannemuehler MJ. 2020. Temporal dynamics of chronic inflammation on the cecal microbiota in IL-10−/− mice. Front Immunol 11:585431. doi:10.3389/fimmu.2020.585431. 33664728 PMC7921487

[80] Büchler G, Wos-Oxley ML, Smoczek A, Zschemisch NH, Neumann D, Pieper DH, Hedrich HJ, Bleich A. 2012. Strain-specific colitis susceptibility in IL10-deficient mice depends on complex gut microbiota-host interactions. Inflamm Bowel Dis 18:943–954. doi:10.1002/ibd.21895. 22238116

[81] Yang I, Eibach D, Kops F, Brenneke B, Woltemate S, Schulze J, Bleich A, Gruber AD, Muthupalani S, Fox JG, Josenhans C, Suerbaum S. 2013. Intestinal microbiota composition of interleukin-10 deficient C57BL/6J mice and susceptibility to *Helicobacter hepaticus-*induced colitis. PLoS One 8:e70783. doi:10.1371/journal.pone.0070783. 23951007 PMC3739778

[82] Kühn R, Löhler J, Rennick D, Rajewsky K, Müller W. 1993. Interleukin-10-deficient mice develop chronic enterocolitis. Cell 75:263–274. doi:10.1016/0092-8674(93)80068-P. 8402911

[83] Jergens AE, Wilson-Welder JH, Dorn A, Henderson A, Liu Z, Evans RB, Hostetter J, Wannemuehler MJ. 2007. *Helicobacter bilis* triggers persistent immune reactivity to antigens derived from the commensal bacteria in gnotobiotic C3H/HeN mice. Gut 56:934–940. doi:10.1136/gut.2006.099242. 17145736 PMC1994361

[84] Biegert G, El Alam MB, Karpinets T, Wu X, Sims TT, Yoshida-Court K, Lynn EJ, Yue J, Medrano AD, Petrosino J, Mezzari MP, Ajami NJ, Solley T, Ahmed-Kaddar M, Klopp AH, Colbert LE. 2021. Diversity and composition of gut microbiome of cervical cancer patients: do results of 16s rrna sequencing and whole genome sequencing approaches align? J Microbiol Methods 185:106213. doi:10.1016/j.mimet.2021.106213. 33785357 PMC9157377

[85] Props R, Kerckhof F-M, Rubbens P, De Vrieze J, Hernandez Sanabria E, Waegeman W, Monsieurs P, Hammes F, Boon N. 2017. Absolute quantification of microbial taxon abundances. ISME J 11:584–587. doi:10.1038/ismej.2016.117. 27612291 PMC5270559

[86] Galazzo G, van Best N, Benedikter BJ, Janssen K, Bervoets L, Driessen C, Oomen M, Lucchesi M, van Eijck PH, Becker HEF, Hornef MW, Savelkoul PH, Stassen FRM, Wolffs PF, Penders J. 2020. How to count our microbes? The effect of different quantitative microbiome profiling approaches. Front Cell Infect Microbiol 10:403. doi:10.3389/fcimb.2020.00403. 32850498 PMC7426659

[87] Limon JJ, Tang J, Li D, Wolf AJ, Michelsen KS, Funari V, Gargus M, Nguyen C, Sharma P, Maymi VI, Iliev ID, Skalski JH, Brown J, Landers C, Borneman J, Braun J, Targan SR, McGovern DPB, Underhill DM. 2019. Malassezia Is associated with Crohn's disease and exacerbates colitis in mouse models. Cell Host Microbe 25:377–388. doi:10.1016/j.chom.2019.01.007. 30850233 PMC6417942

[88] Bashiardes S, Zilberman-Schapira G, Elinav E. 2016. Use of metatranscriptomics in microbiome research. Bioinform Biol Insights 10:19–25. doi:10.4137/BBI.S34610. 27127406 PMC4839964

[89] Davids M, Hugenholtz F, dos Santos VM, Smidt H, Kleerebezem M, Schaap PJ. 2016. Functional profiling of unfamiliar microbial communities using a validated *de novo* assembly metatranscriptome pipeline. PLoS One 11:e0146423. doi:10.1371/journal.pone.0146423. 26756338 PMC4710500

[90] Cox JW, Ballweg RA, Taft DH, Velayutham P, Haslam DB, Porollo A. 2017. A fast and robust protocol for metataxonomic analysis using RNAseq data. Microbiome 5:7. doi:10.1186/s40168-016-0219-5. 28103917 PMC5244565

[91] Parang B, Barrett CW, Williams CS. 2016. AOM/DSS model of colitis-associated cancer. Methods Mol Biol 1422:297–307. doi:10.1007/978-1-4939-3603-8_26. 27246042 PMC5035391

[92] Tanaka T, Kohno H, Suzuki R, Yamada Y, Sugie S, Mori H. 2003. A novel inflammation-related mouse colon carcinogenesis model induced by azoxymethane and dextran sodium sulfate. Cancer Sci 94:965–973. doi:10.1111/j.1349-7006.2003.tb01386.x. 14611673 PMC11160237

[93] Suzuki R, Kohno H, Sugie S, Nakagama H, Tanaka T. 2006. Strain differences in the susceptibility to azoxymethane and dextran sodium sulfate-induced colon carcinogenesis in mice. Carcinogenesis 27:162–169. doi:10.1093/carcin/bgi205. 16081511

[94] Gordo I. 2019. Evolutionary change in the human gut microbiome: from a static to a dynamic view. PLoS Biol 17:e3000126. doi:10.1371/journal.pbio.3000126. 30730933 PMC6366738

[95] Sousa A, Frazão N, Ramiro RS, Gordo I. 2017. Evolution of commensal bacteria in the intestinal tract of mice. Curr Opin Microbiol 38:114–121. doi:10.1016/j.mib.2017.05.007. 28591676

[96] Voolstra CR, Ziegler M. 2020. Adapting with microbial help: microbiome flexibility facilitates rapid responses to environmental change. Bioessays 42:e2000004. doi:10.1002/bies.202000004. 32548850

[97] Ramiro RS, Durão P, Bank C, Gordo I. 2020. Low mutational load and high mutation rate variation in gut commensal bacteria. PLoS Biol 18:e3000617. doi:10.1371/journal.pbio.3000617. 32155146 PMC7064181

[98] Phifer-Rixey M, Nachman MW. 2015. Insights into mammalian biology from the wild house mouse *Mus musculus*. Elife 4:e05959. doi:10.7554/eLife.05959. PMC4397906 25875302

[99] Shneider MM, Buth SA, Ho BT, Basler M, Mekalanos JJ, Leiman PG. 2013. PAAR-repeat proteins sharpen and diversify the type VI secretion system spike. Nature 500:350–353. doi:10.1038/nature12453. 23925114 PMC3792578

[100] Caruso R, Mathes T, Martens EC, Kamada N, Nusrat A, Inohara N, Núñez G. 2019. A specific gene-microbe interaction drives the development of Crohn's disease-like colitis in mice. Sci Immunol 4:eaaw4341. doi:10.1126/sciimmunol.aaw4341. 31004013 PMC8882361

[101] Chin CS, Alexander DH, Marks P, Klammer AA, Drake J, Heiner C, Clum A, Copeland A, Huddleston J, Eichler EE, Turner SW, Korlach J. 2013. Nonhybrid, finished microbial genome assemblies from long-read SMRT sequencing data. Nat Methods 10:563–569. doi:10.1038/nmeth.2474. 23644548

[102] Seemann T. 2014. Prokka: rapid prokaryotic genome annotation. Bioinformatics 30:2068–2069. doi:10.1093/bioinformatics/btu153. 24642063

[103] Kanehisa M, Sato Y, Morishima K. 2016. BlastKOALA and GhostKOALA: KEGG tools for functional characterization of genome and metagenome sequences. J Mol Biol 428:726–731. doi:10.1016/j.jmb.2015.11.006. 26585406

[104] Chen C, Khaleel SS, Huang H, Wu CH. 2014. Software for pre-processing Illumina next-generation sequencing short read sequences. Source Code Biol Med 9:8. doi:10.1186/1751-0473-9-8. 24955109 PMC4064128

[105] Schmieder R, Edwards R. 2011. Quality control and preprocessing of metagenomic datasets. Bioinformatics 27:863–864. doi:10.1093/bioinformatics/btr026. 21278185 PMC3051327

[106] Galperin MY, Makarova KS, Wolf YI, Koonin EV. 2015. Expanded Microbial genome coverage and improved protein family annotation in the COG database. Nucleic Acids Res 43:D261–D269. doi:10.1093/nar/gku1223. 25428365 PMC4383993

[107] Love MI, Huber W, Anders S. 2014. Moderated estimation of fold change and dispersion for RNA-seq data with DESeq2. Genome Biol 15:550. doi:10.1186/s13059-014-0550-8. 25516281 PMC4302049

[108] Caporaso JG, Kuczynski J, Stombaugh J, Bittinger K, Bushman FD, Costello EK, Fierer N, Peña AG, Goodrich JK, Gordon JI, Huttley GA, Kelley ST, Knights D, Koenig JE, Ley RE, Lozupone CA, McDonald D, Muegge BD, Pirrung M, Reeder J, Sevinsky JR, Turnbaugh PJ, Walters WA, Widmann J, Yatsunenko T, Zaneveld J, Knight R. 2010. QIIME allows analysis of high-throughput community sequencing data. Nat Methods 7:335–336. doi:10.1038/nmeth.f.303. 20383131 PMC3156573

[109] Li H, Durbin R. 2009. Fast and accurate short read alignment with Burrows-Wheeler transform. Bioinformatics 25:1754–1760. doi:10.1093/bioinformatics/btp324. 19451168 PMC2705234

