# Erratum for Proctor et al., “Resources To Facilitate Use of the Altered Schaedler Flora (ASF) Mouse Model To Study Microbiome Function”

**DOI:** 10.1128/msystems.01205-22

**Published:** 2023-02-07

**Authors:** Alexandra Proctor, Shadi Parvinroo, Tanner Richie, Xinglin Jia, Sonny T. M. Lee, Peter D. Karp, Suzanne Paley, Aleksandar D. Kostic, Joseph F. Pierre, Michael J. Wannemuehler, Gregory J. Phillips

## ERRATUM

Volume 7, no. 5, e00293-22, 2022, https://doi.org/10.1128/mSystems.00293-22. In Table S1 in the supplemental material, the gBlock sequence for ASF# 492 is given incorrectly. The actual sequence is given in the revised file below.

10.1128/msystems.01205-22.1 TABLE S1 *groEL* gBlock sequences. Download Table S1, DOCX file, 0.02 MB. Copyright © 2023 Proctor et al. 2023 Proctor et al. https://creativecommons.org/licenses/by/4.0/ This content is distributed under the terms of the Creative Commons Attribution 4.0 International license.

