# Supplementary figures and images for: Resources to Facilitate Use of the Altered Schaedler Flora (ASF) Mouse Model to Study Microbiome Function
Source: mSystems. 2022 Aug 15;7(5):e00293-22. doi: 10.1128/msystems.00293-22 (PMC9600240; doi:10.1128/msystems.00293-22)

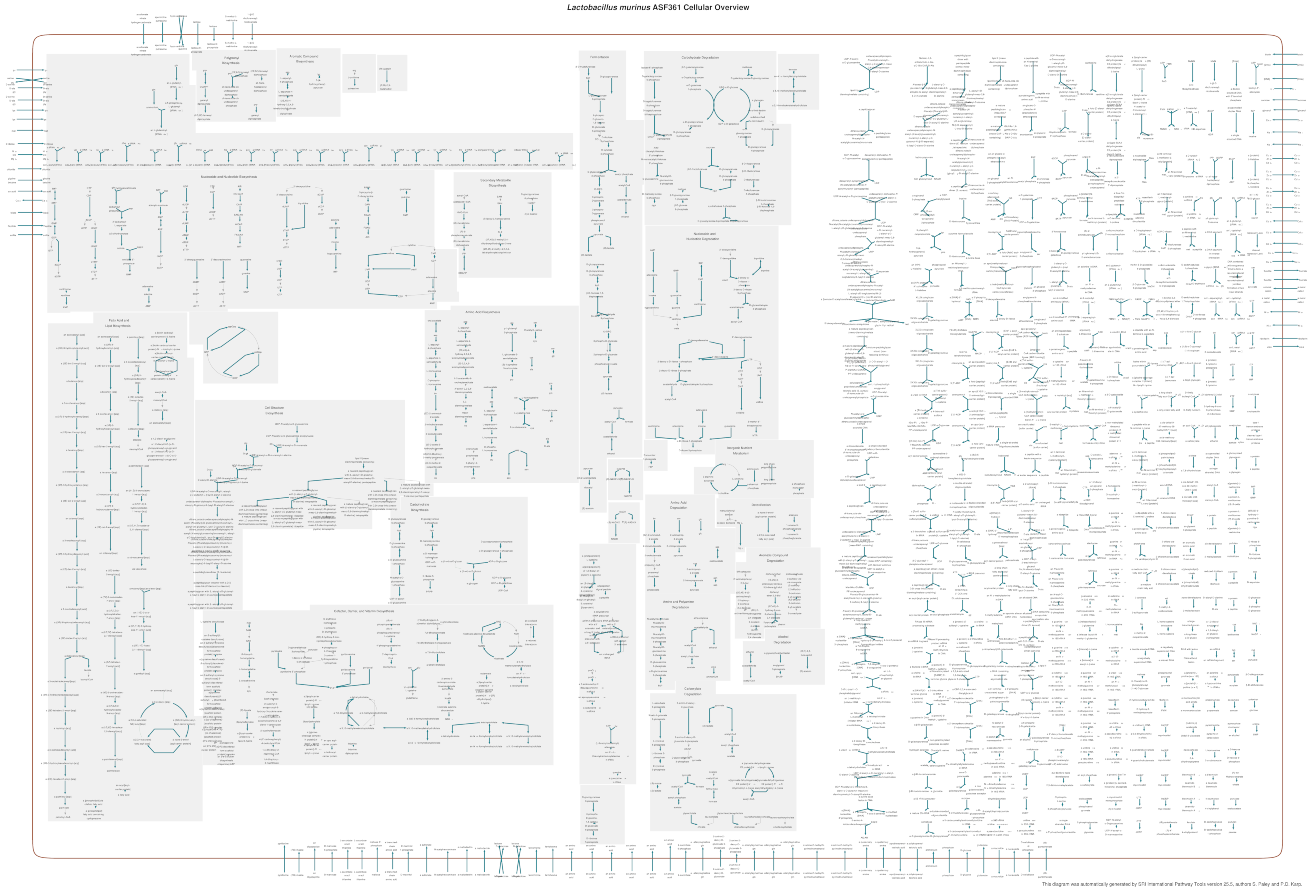

Supplement: FIG S1 [file msystems.00293-22-s0003.tif]

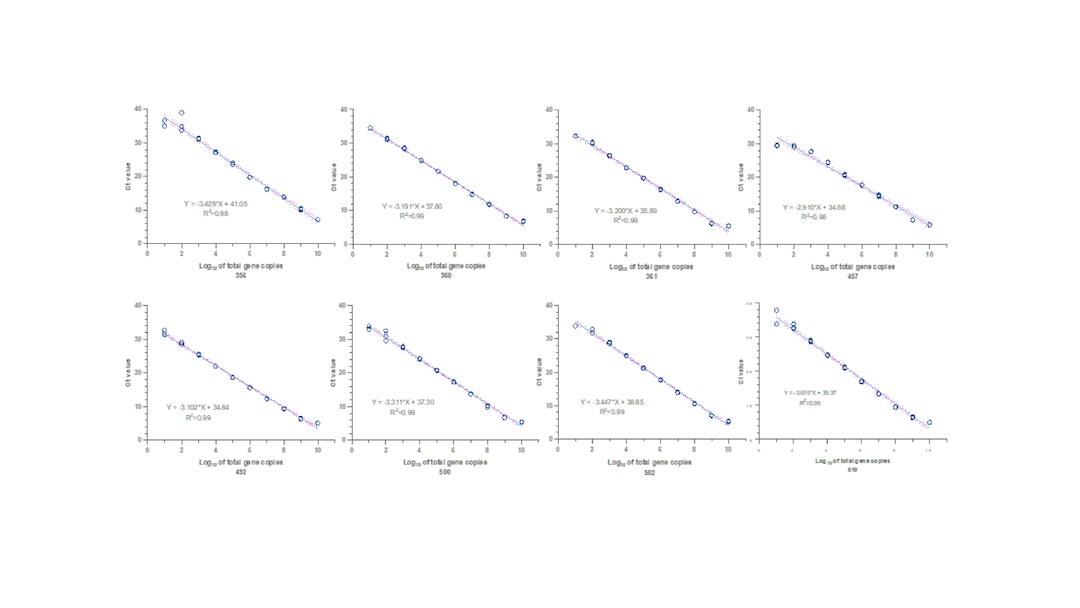

Supplement: FIG S2 [file msystems.00293-22-s0004.tif]

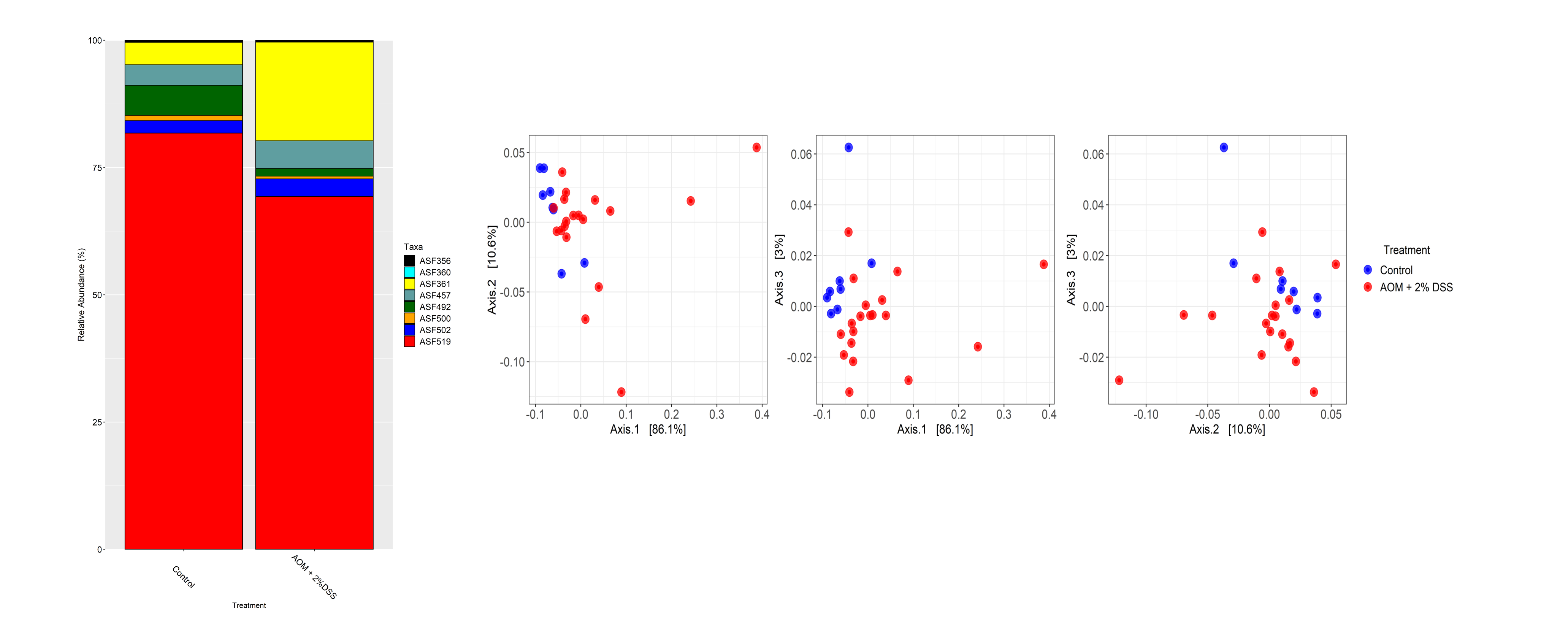

Supplement: FIG S3 [file msystems.00293-22-s0005.tif]
