# Supplementary material for: Resources to Facilitate Use of the Altered Schaedler Flora (ASF) Mouse Model to Study Microbiome Function
Source: mSystems. 2022 Aug 15;7(5):e00293-22. doi: 10.1128/msystems.00293-22 (PMC9600240; doi:10.1128/msystems.00293-22)
Supplement: TABLE S2 [file msystems.00293-22-s0002.docx]

**Table S2. Colonization of the ASF into 129S6/SvEv mice**

| Genotype of germ free (GF) mice | 129S6/SvEv wild type (WT) and IL-10 knockout (IL-10^-/-^/KO) |
| --- | --- |
| Source of mice | National Gnotobiotic Rodent Resource Center at University of North Carolina |
| Housing and feed | Sterile isolator, given sterile water and irradiated mouse chow |
| Protocol for colonization of ASF into GF mice | C3H/HeNTac mice harboring the ASF were euthanized via CO_2_ asphyxiation with secondary exsanguination via cardiac puncture. The ceca were ligated and resected so that the contents were maintained under strict anaerobic conditions. The cecal contents were resuspended in sterile BHI broth and used to immediately inoculate GF mice via oral gavage with the cecal slurry inside the sterile isolator. Sterile technique was used in a disinfected biosafety hood were utilized for fecal collection. The mice were monitored over several weeks for contamination and ASF colonization using standard aerobic culture on BHI media and end-point PCR with primers specific for each of the ASF (Table 1). |
