# Supplementary material for: Erratum for Proctor et al., “Resources To Facilitate Use of the Altered Schaedler Flora (ASF) Mouse Model To Study Microbiome Function”
Source: mSystems. 2023 Feb 7;8(2):e01205-22. doi: 10.1128/msystems.01205-22 (PMC10134810; doi:10.1128/msystems.01205-22)
Supplement: TABLE S1 [file msystems.01205-22-s0001.docx]

**TABLE S1 *groEL* gBlock sequences**

| **ASF #** | **gBlock sequence** |
| --- | --- |
| 356 | CACTTGCTACATTAGTTGTAAATAAATTAAGAGGTACTATTAATTGTGTTGCAGTGAAAGCTCCTGGATATGGTGAAAGAAGAAAAGCACAAATTGCTGATATTGCAGCTTTAACAGGTGCTCAAGTAATTTCTGATGAGTTAGGTATTGATTTAAAAGAAGCTACTATTGATATGCTTGGTCATGCAAGAACAGTAAGAGTAGAAAAAGAATTGACAATTATTGCTGATGGTGCTGGAAACAAAGAAGATATTCAAGGCAGAATTAACCAAATCAAAGCACAATTGGAAGAAACAACATCTGATTTTGATAAAGAAAAATTACAAGAAAGATTAGCAAAATTATCTGGTGGTGTTGCTGTTATTAAAGTTGGTGCTGCAACAGAAACAGAAATGAAAGAGAAGAAATTAAGAATGGAAGATGCTTTAGCAGCTACAAGAGCAGCAGTAGAAGAAGGTATTGTTGCTGGTGGTGGTGCTG |
| 360 | GTTTGGCTAAGTTAACTGGTGGTGTAGCTGTTATCCACGTTGGTGCTGCTACTGAAACCGAATTGAAGGAACGTCGTTACAGAATTGAAGATGCTTTGAACTCAACTCGTGCCGCTGTTGACGAAGGTTATGTTGCAGGTGGTGGTACTGCCTTAGTAAATGTTGAAAGCGCAGTTCGTGAAGCTAAGGGTGACACTCCAGATGAACAAACTGGTATCAATATTGTCTTGAGAGCACTTTCTGCACCAGTTCGTCAAATTGCTGACAACGCTGGTAAGGATGGTTCAGTCATCTTGAACAAGCTTGAAAATGAAAAGCCAGAAATTGGTTACAATGCTGCAACTGATGAATGGGTAAACATGGTTGATGCTGGTATCATCGACCCAACTAAGGTAACTCGTACCGCCCTTCAAAATGCTGCTTCAATTGCAGGACTTCTCCTTACTACTGAAGCTGTAGTTGCAGATATTCCTGAAGACAAGCCACAAAACCCAGCTG |
| 361 | GACATTCAATGTGGTAGCCGTTAAAGCTCCAGGTTTTGGTGATCGTCGTAAAGAAATGTTACAAGATATCGCTGTTTTAACTGGTGCAACTGTGATCACAGATGATCTCGGTCTTCAATTAAAGGACACGACTTTAGATCAATTAGGGACAGCTGGTCGTGTAACAGTTACAAAAGAAAATACAACGATCGTCGAAGGTGCTGGAGATAAAGCACAGATCGCAGAACGTGTTGAACAACTCAAAAAGCAGATCGCTGAAACGACCTCTGAATTTGATAAAGAAAAACTCCAAGAACGTTTAGCTAAATTAGCTGGTGGGGTAGCGGTTATCAAAGTCGGGGCTGCTACTGAGACTGAATTAAAAGAACGCAAATACCGGATCGAAGACGCTTTGAATGCTACACGCGCAGCTGTTGAAGAAGGTTTTGTTCCTGGTGGTGGGACAGCTTTGATCGATGTGATCGATGATGTAGCTACTTTGAGTGAAGCTGGTGATGTAC |
| 457 | AACTGTGTGGCTGTAAAAGCTCCTGGCTTTGGCGACAGAAGAAAAGAAATGCTTAAAGATATTGCAGTATTAACTGGCGGTCAGGTTATTACTGATGATTTAGGTATTAAAATAGATAGTGTTGAACTTGAAGATTTAGGCAGAGCAAAAAAAGTTGTTATTGATAAAGATAACACAACAATTGTTGAAGGAGCAGGTAATACTGACGATATTAAAGCAAGAGTTACTCAAATCAAAAAACAAATTGAAGATACTACAAGTGACTATGACAGAGAAAAACTTCAAGAACGCCTTGCTAAATTAATTGGTGGTGTAGCAGTTATTAAAGTTGGTGCTGCAACAGAAACTGAAATGAAAGAGAAAAAACATAGAGTGGAAGATGCTCTTGCAGCTACAAAAGCAGCTGTTGAAGAAGGCATTGTTCCTGGAGGCGGTGTTGCATTAGTAAGAACTGTTTCAGCTCTTAAAAATATAAAAGTTTCTC |
| 492 | GCTGGATCTTGTAGAAGGTATGCAGTTTGACCGTGGTTATATTTCCGCTTATATGGCAACGGATATGGATAAAATGGAAGCTGTTTTAGAAGATCCGTATATTTTAATTACAGATAAAAAGATTTCCAATATTCAGGAAATTCTTCCGTTACTGGAGCAGGTTGTAAAGACAGGCGCAAAACTGCTTATTATTGCAGAAGACGTAGAAGGCGAAGCGCTTACAACATTAATCGTTAACAAACTGCGTGGTACATTTAACGTAGTGGCAGTCAAAGCTCCTGGTTATGGTGACAGAAGAAAAGCTATGCTGGAAGATATTGCAATACTGACCGGCGGACAGGTCATTTCTTCCGAACTGGGCTTGGAATTAAAAGATGCCGAATTAGAGCAGTTAGGACGTGCAAAATCCGTAAAAGTACAGAAAGAAAATACGGTAATTGTAGACGGTATGGGTGAAAAATCTGCTATTGATGCCAGAATCAGCCAGATTAAGAGCCAGATCGAAGAA |
| 500 | GACCGGGGCTACATCACCCCCTATATGGTCACCGACACCGAAAAGATGGTGGCCGAGCTGGACGACGCCCTCATCCTCATTACCGACAAGAAGATCTCCAACATTCAGGAGCTGCTGCCCATTCTGGAGCAGGTGGTCCAGTCCGGCAAGAAGCTGCTGATTATTGCCGAGGACGTGGAGGGCGACGCCCTGTCCACCCTGATTGTCAACCGTCTCCGGGGCACCCTGAATGTGGTGTGCGTCAAGGCTCCCGGCTTCGGTGACCGCCGCAAGGAGATGCTTCAGGATATCGCCATCCTCACCGGCGGCGAGGTCATCTCCGCCGACGTGGGCCTGGAGCTGAAGGAGGCTCAGATGAACATGCTGGGCTCCGCCCGCCAGGTGAAGATCACCAAGGAGAACACCACCATCGTCAACGGCGCGGGCTCCACCGAGGAGATCAAGGCCCGCATCGGCCAGATCAAGAGCC |
| 502 | CGCATATATGGCGACAGATATGGAGAAGATGGAAGCGGTTCTGGAAGATCCGTACATCCTCATCACAGATAAGAAGATTTCTAATATCCAGGATATCCTTCCGCTTCTGGAGCAGGTGGTACAGTCCGGCGCAAGGCTTCTGATCATCGCAGAGGATATTGAGGGAGAGGCGCTGACCACTCTGATCGTGAACAAGCTGCGCGGAACCTTCAATGTAGTAGCTGTAAAGGCTCCGGGATACGGCGACAGAAGAAAAGAAATGCTGCAGGATATCGCGATCCTGACAGGTGGACAGGTGATCTCCGAAGAACTTGGCCTGGATCTGAAGGATACCACCATGGATCAGCTTGGACGTGCGAAGTCCGTCAAGGTACAGAAGGAAAATACCGTAATCGTAGACGGCTCCGGCGACAAGCAGGCGATTGCCGACAGAGTGGCTCAGATCAAGAAGGGCATCGAAGAGACCACTTCTGATTTT |
| 519 | AACGTTACAGCAGGTGCTAACCCGATGGACCTGAAACGCGGTATCGACAAAGCCGTTGCCAAAGTGGTTGAAAAGATCGCTGACCAGGCTGAAGAAGTAGGCGACCAGTTCGAAAAGATCGAACACGTAGCTAAGATCTCTGCTAACGGTGACGAAATGATCGGTAAACTGATTGCTGAAGCTATGCAGAAAGTGAAGAAAGAAGGCGTTATCACAGTGGAAGAAGCAAAAGGTACTGAAACTACAGTTGACGTAGTAGAAGGTATGCAGTTCGACCGTGGTTACATCTCTCCGTACTTCGTAACCAACACAGAAAAGATGGAATGCGAGATGGAAAATCCGTATATCCTGATCTACGACAAGAAGATCTCTGTATTGAAGGACTTGCTTCCTATCCTGGAACCGGCTGTTCAGAGCGGTCGTCCGCTGTTGATCATCGCAGAAGATATCGACAGCGAAGCATTGGCTACATTGGTTGTAAACCGTCTGCGTGGTTCTTTGAAG |
